# Lipid Emulsion as a Treatment for Non-Local Anesthetic Poisoning in Animals: A Systematic Review and Meta-Analysis of Survival and Neurological Outcomes

**DOI:** 10.3390/vetsci13101059

**Published:** 2026-10-08

**Authors:** Alex M. Lynch, Alex Lillis, Adalynn Mulvey-Rose, David C. Provost, Suzie Schoolfield, David C. Dorman

**Affiliations:** 1 College of Veterinary Medicine, North Carolina State University, Raleigh, NC 27606, USA; arlillis@ncsu.edu (A.L.); addymaymr@gmail.com (A.M.-R.); david_dorman@ncsu.edu (D.C.D.); 2 William Rand Kenan, Jr. Library of Veterinary Medicine, North Carolina State University Libraries, Raleigh, NC 27607, USA; dave_provost@ncsu.edu

**Keywords:** systematic review, meta-analysis, intravenous lipid emulsion, poisoning, veterinary, neurotoxicity, animal models

## Abstract

A prior scoping review indicates that veterinarians use intravenous lipid emulsion (ILE) to treat poisoned animals, especially animals exposed to suspect neurotoxicants. The benefit of ILE in these cases remains uncertain. We conducted a systematic review and post hoc meta-analysis examining the use of ILE therapy in experimental animal models of neurotoxicosis and survival. We evaluated the relative risk of mortality using a random effects model. Neurological outcomes, including neuropathology, neurological signs, behavior, and anesthetic recovery, were assessed qualitatively. Of 3937 references screened, 38 met criteria for inclusion. In the global meta-analysis considering all toxicants and animal species, ILE treatment was associated with a lower risk of death (RR = 0.67; 95% CI, 0.48–0.93; *p* = 0.007). Possible publication bias was detected (Egger’s test, *p* = 0.005), and between-study heterogeneity was high (I^2^ = 74.5%). In a subgroup analysis excluding swine studies, ILE treatment was associated with reduced mortality (RR = 0.52; 95% CI, 0.33–0.82; *p* = 0.005); however, possible publication bias and high heterogeneity were present. A qualitative evaluation of neurological outcomes revealed heterogeneous results, incomplete reporting of studies resulting in risk of bias concerns in multiple domains, and small sample sizes. Exploratory meta-analysis of ILE demonstrated a reduction in mortality. Results were characterized by high heterogeneity, possible publication bias, methodological limitations, and very low certainty of evidence. Larger, adequately powered controlled clinical studies in naturally poisoned veterinary patients are needed.

## Simple Summary

Poisoning from chemicals that affect the brain and nervous system can be life-threatening in animals. Veterinarians sometimes use a fat-based treatment, known as intravenous lipid emulsion, to help poisoned animals recover, but it is still unclear how well this treatment works. The goal of this study was to review and combine the results of many animal studies to better understand whether this treatment improves survival and recovery in poisoned animals. We critically evaluated 36 experimental studies and two clinical trials involving different poisons and animal species to determine whether animals treated with a lipid emulsion were less likely to die or develop neurological effects after poisoning. The most consistent positive finding was faster recovery from anesthesia or sedation. This finding was based on two small experimental studies. When mortality studies were combined, animals treated with a lipid emulsion had a lower risk of death than control animals. However, results varied substantially among studies, evidence of publication bias was detected, and the overall certainty of evidence for a survival benefit was very low. Some studies suggested possible improvement in certain signs of illness, but the findings were inconsistent, and many studies had important weaknesses, such as small numbers of animals and incomplete reporting. Overall, current evidence is insufficient to determine whether lipid emulsion treatment consistently improves survival or neurological recovery in poisoned animals. Most of the available evidence came from experimental animal models that differ substantially from naturally occurring poisoning in veterinary patients, further limiting the clinical application of these findings. The study highlights the need for better quality research before clear treatment recommendations can be made.

## 1. Introduction

Intravenous lipid emulsion (ILE) formulations contain triglycerides derived from soybean, coconut, olive, and fish oils and are commercially available in concentrations of 10%, 20%, and 30%. Clinically, ILEs are widely used in parenteral nutrition to prevent essential fatty acid deficiency [1]. ILEs are also used as delivery vehicles for lipid-soluble medications, including propofol. Since the mid-2000s, ILE therapy has gained recognition as a treatment for local anesthetic systemic toxicity (LAST), including local anesthetic-induced cardiac arrest. Reported applications in LAST have involved toxicities associated with agents such as bupivacaine, cocaine, lidocaine, and mepivacaine. This therapeutic approach, often referred to as “lipid rescue” or “lipid resuscitation” therapy, has been evaluated in several recent reviews [2,3,4].

In human medicine, ILE has also been used in the management of poisonings caused by non-local anesthetic agents including tricyclic antidepressants, calcium channel blockers, beta blockers, antipsychotics, and other drugs [5,6,7,8,9]. The evidence base supporting the use of ILE in non-LAST poisoning often relies on successful case reports or animal studies, limiting evidence-based conclusions regarding the efficacy of ILE in human poisoning cases [10,11,12,13,14].

Despite increasing clinical use, the precise mechanisms through which ILE improves outcomes in poisoning cases remain incompletely characterized. Several complementary theories have been proposed [8,15,16]. Some theories propose that the intravascular lipid phase sequesters lipophilic toxicants from target tissues such as the heart and central nervous system (lipid sink theory), while others propose that ILE droplets absorb lipophilic toxicants and shuttle and redistribute the bound toxicant into storage compartments with lower sensitivity, such as skeletal muscles and adipose tissue (lipid shuttle theory), thereby reducing tissue bioavailability and toxicity [15,16]. Thus, the lipid sink hypothesis emphasizes removal of free toxicant from target tissues into the intravascular lipid phase, whereas the lipid shuttle hypothesis extends this concept by proposing subsequent transport and redistribution of the toxicant to less sensitive tissues. In addition, ILE may exert direct cardiometabolic effects by supplying free fatty acids as an energy substrate to myocardial tissue, particularly during toxin-induced cardiovascular compromise [16,17,18]. Experimental evidence has also suggested that ILE may improve cardiac contractility, enhance mitochondrial fatty acid metabolism, and modulate intracellular calcium channels, thereby contributing to improved hemodynamic function independent of toxicant sequestration [16,17,18].

The efficacy and safety of ILE in veterinary clinical toxicology remain incompletely characterized. This systematic review builds on a prior scoping review conducted by the authors [19], which examined the use of ILE in the management of non-LAST in animals. The scoping review included experimental studies, randomized clinical trials, case series, and case reports involving species such as dogs, cats, guinea pigs, goats, birds, and sea turtles. Among published veterinary case reports, the most common indications for ILE therapy we found included poisonings involving permethrin, ivermectin, baclofen, bromethalin, moxidectin, and other neurotoxic agents [19]. Consistent with these observations, a recent multicenter case series described the use of ILE in dogs and cats with suspected neurotoxicosis [20].

The present systematic review evaluates the use of lipid emulsions in animals and considers evidence from randomized clinical trials and experimental studies. Our review considers two main outcomes of interest—survival and neurological effects. Neurological outcomes were considered alongside survival because toxicant exposure may result in substantial neurological morbidity in animals that survive and improvement in neurological function represents a clinically relevant treatment outcome. The present review focuses on post-toxicant exposure with ILE to more closely mimic veterinary clinical applications, as veterinary patients are typically treated after a delay between toxicant exposure and ILE administration. This study addressed the following review question: Does the use of ILE therapy in isolation or combined with other therapies decrease either the incidence or severity of neurological disease or mortality in vertebrate animals exposed to a toxicant?

This systematic review and meta-analysis provide a novel synthesis of the experimental and controlled clinical evidence evaluating ILE following toxicant exposure in animals, with specific consideration of survival and neurological outcomes. Important gaps in this evidence base include uncertainty regarding the consistency of treatment effects across toxicants and species, the translational relevance of experimental models to veterinary patients, and the methodological quality of the available studies. Addressing these gaps is important for determining whether evidence supports the clinical use of ILE for non-local anesthetic poisoning in veterinary medicine.

## 2. Materials and Methods

### 2.1. Protocol and Registration

This study was conducted according to the Preferred Reporting Items for Systematic Reviews and Meta-Analyses guidelines [21]. The PRISMA checklist was followed for each section of the meta-analysis (https://www.prisma-statement.org/ accessed on 5 October 2026). Table S1 provides the completed PRISMA checklist for this study. The protocol was prospectively designed and made available via Open Science Framework (see https://osf.io/9uk7a/files/xf9nr accessed on 5 October 2026). The registered protocol specified searching the gray literature, including conference proceedings and dissertations. During conduct of the review, eligibility was restricted to peer-reviewed publications, and gray literature was not included. The protocol detailed the research question and outcomes of interest, outlined a search strategy and the process of data extraction, and provided criteria for rating the quality of evidence. The protocol did not designate a single primary outcome. The two prespecified outcome domains were (1) survival or survival-related endpoints and (2) the incidence or severity of neurological outcomes. For purposes of evidence synthesis, neurological outcomes were subsequently categorized as neuropathology, neurological signs, behavior, and recovery from anesthesia or sedation. The registered protocol specified that quantitative synthesis would be performed when appropriate but did not prespecify the specific meta-analytic model or subgroup analyses subsequently performed. These analyses were developed after evaluation of the eligible evidence and should therefore be considered exploratory.

### 2.2. Search Strategy

Sources included the following databases: PubMed, Web of Science, and CAB Abstracts. An information specialist (DP) with training, expertise, and familiarity with developing and performing systematic review literature searches designed and conducted the search. Supplemental File S1 provides the search strategy for the search, including search syntaxes for all databases. The search was peer reviewed by an information specialist prior to conduct of the search. The literature search was initially conducted on 23 October 2025.

### 2.3. Study Eligibility and the PICO Framework

This study used the following PICO framework for the conduct of the systematic review:

- Population: Vertebrate animals without sex, age, or breed restrictions.
- Intervention: Any ILE therapy, irrespective of dose, route of administration, or duration of treatment, given after a toxicant exposure.
- Comparator: Vertebrate animals of the same species exposed to a toxicant and treated with any non-lipid emulsion drug treatment compared to placebo-treated animals or other dosage of the same lipid emulsion treatment (dose response).
- Outcome: Clinical outcomes of interest include survival (and survival endpoints) and incidence and severity of neurological signs (e.g., central nervous system depression, seizures, muscle tremors).
- Additional criteria: Studies included original data (commentaries and editorials were excluded). Studies evaluating ILE in the management of local anesthetics were excluded. Although the registered protocol specified inclusion of the gray literature, the completed review was restricted to peer-reviewed articles. Conference proceedings, dissertations, and other gray literature were therefore excluded to maintain a reproducible search of peer-reviewed studies with sufficient methodological information for eligibility and risk of bias assessment. Eligible study designs included randomized clinical trials (RCTs), non-randomized intervention trials, and controlled experimental studies. Experimental studies were defined as in vivo studies in which animals were exposed to a toxicant and subsequently administered ILE, with outcomes compared with a concurrent control or alternative treatment group or across ILE dose groups. Studies were not restricted by either publication date or language.

Eligible studies were aligned with the PICO framework described above. For purposes of study eligibility, we did not require the toxicant to meet a predefined mechanistic definition of a neurotoxicant. Instead, neurotoxicosis was operationally defined as toxicant exposure associated with one or more neurological outcomes, including alterations in neurological examination findings, seizures, tremors, behavior, neuropathology, or recovery from anesthesia or sedation. Studies evaluating survival were also eligible irrespective of whether neurological signs were reported.

All search results were imported or manually entered into Covidence (https://www.covidence.org/home accessed on 5 October 2026), and this software was used to eliminate duplicate citations before evaluating the eligibility of the citations. Exclusion criteria included human studies, non-vertebrate animal species, ILE given as a drug vehicle, ILE given prior to toxicant exposure, local anesthetics, no control or dose response, no assessment of either survival or neurological signs, case reports or case series lacking a control, and cohort studies lacking a comparison group.

Following peer review, studies excluded during initial screening were re-evaluated against the prespecified eligibility criteria. Three additional experimental studies containing eligible survival outcomes were identified and included, and all mortality meta-analyses were repeated.

### 2.4. Study Screening

References retrieved from the literature search were screened for relevance and eligibility against the evidence selection criteria using Covidence (https://www.covidence.org/home). Title and abstract screening were initially performed, followed by full-text screening of studies that were initially identified. Each citation was independently screened by two reviewers to determine whether a study met the selection criteria for inclusion that reflect the PICO statement, with additional considerations listed above. 

### 2.5. Data Extraction

Data were collected and recorded from included studies by one member of the review team and checked by a second member for completeness and accuracy. Data extraction was completed using study-specific tables. Discrepancies in data extraction were resolved through discussion. The extracted data were used to summarize study designs and findings and/or to conduct statistical analyses. Extracted information included study characteristics, exposure details, dose, duration, and frequency of ILE used, outcomes, and measures of effect. Extraction of graphical data relied on DigitizeIt version 2.5.1.

### 2.6. Quality Assessment

Methodological quality and risk of bias were evaluated using signaling questions provided in the SYRCLE tool [22]. Risk of bias in individual studies was assessed by members of the review team working in pairs independently of each other using Covidence systematic review software. Coauthors of studies were excluded from evaluating their publications for risk of bias. Each member of the team evaluated each study across the following seven risk of bias domains: random sequence generation; allocation sequence; blinding of participants and personnel; blinding of outcome assessment; incomplete outcome data; selective reporting; and other sources of bias. Risk of bias ratings for each domain were low risk of bias, unknown risk of bias, or high risk of bias. Information or study procedures that were unreported were assumed not to have been conducted, resulting in an assessment of “unknown” risk of bias. Study authors were not contacted for missing data.

Inter-rater reliability of the risk of bias assessments was evaluated using Cohen’s kappa (κ). The two reviewers independently classified each domain as low, high, or unclear risk of bias. Cohen’s kappa was calculated using the three categorical ratings, both separately for each risk of bias domain and across all domain assessments combined, prior to resolution of disagreements through consensus. Inter-rater agreement for risk of bias assessments was 75.0%, with an overall Cohen’s κ of 0.51 (95% CI, 0.43–0.59), indicating moderate agreement.

### 2.7. Method of Analysis

JMP^®^ Student Edition version 19.1.4 (JMP Statistical Discovery LLC, Cary, NC, USA) was used to calculate inter-rater reliability. MedCalc version 23.5.5 (MedCalc Software Ltd., Ostend, Belgium) was used for all other statistical analyses. Forest plot analysis was used to look at interstudy heterogeneity for clinical outcomes. Results were reported as risk ratios (RR) comparing the incidence of a clinical outcome (e.g., mortality) in experimental groups to control groups. A value of 0.5 was added to all cells when zeros in an incidence table led to computational errors [23]. A random effect Mantel–Haenszel model (95% CI) was used to determine effect sizes between studies. Some studies included multiple arms where controls were shared between experimental groups. In this case, results were pooled. Statistical heterogeneity was assessed using the I^2^ statistic. I^2^ values of approximately 25%, 50%, and 75% were interpreted as indicating low, moderate, and high heterogeneity, respectively [24]. Random effect models were preferred over fixed effect models due to the evidence of high heterogeneity between trials when a fixed effect model was used. Risk ratios were selected because mortality was a directly observed binary outcome and the RR provides a readily interpretable measure of mortality risk in ILE-treated animals relative to control animals. Exploratory analyses included toxicant-specific subgroup analyses for tricyclic antidepressants and organophosphate pesticides and a sensitivity analysis excluding swine studies. Funnel plot asymmetry/small study effects were assessed using Egger’s test.

### 2.8. Evidence Synthesis

Assessment of the quality, quantity, and consistency of evidence across studies was independently performed by at least two members of the review team using the Grading of Recommendations, Assessment, Development, and Evaluation (GRADE) approach [25]. In brief, studies on a particular outcome were initially grouped by key study design features, and each grouping of studies was given an initial confidence rating based on those features. The initial confidence rating ranges from 0 to 4, with one point given for each of the following features: (a) controlled exposure; (b) exposure before outcome; (c) individual outcome data; and (d) comparison group used. Several factors were then considered to determine whether the initial rating should be either downgraded or upgraded. Factors that could downgrade the rating included risk of bias, indirectness (use of surrogate outcomes), inconsistency (heterogeneity), imprecision (wide confidence intervals around the effect), and publication bias. Factors that could upgrade the rating included large magnitude of effect, dose response, and accounting for plausible confounders. To obtain the final GRADE score for a given outcome, points were deducted from the initial GRADE score based on criteria related to the following five categories: quality, indirectness, consistency, precision, and publication bias. After a final confidence rating was determined, the rating was translated into a level of evidence using the following scheme: final score ≤ 1: very low; 2: low; 3: moderate; ≥4: high. Evidence profiles and summary of findings tables were created using a customized form.

## 3. Results

### 3.1. Results of the Search

**Figure 1:**
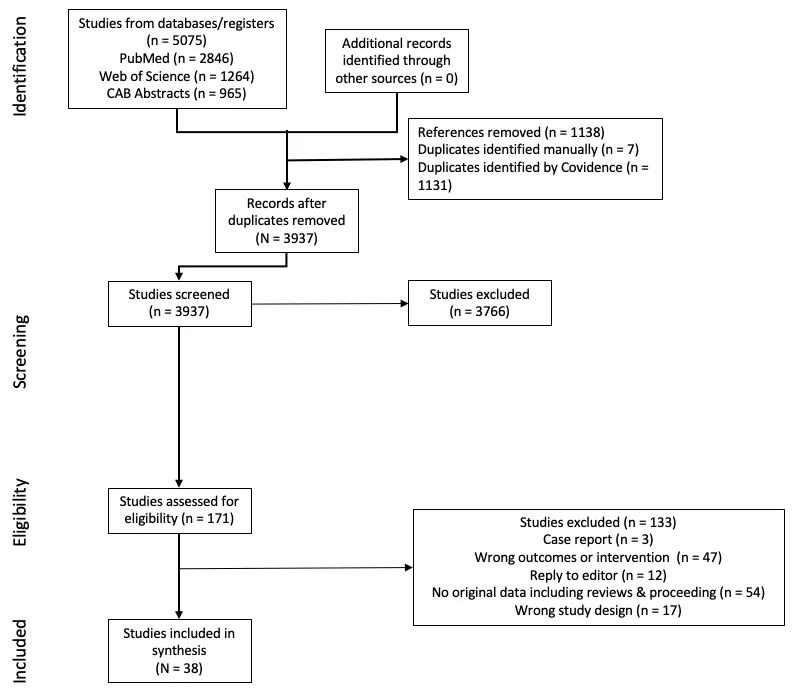
PRISMA diagram.

The search strategy identified 5075 citations, of which 1138 were duplicate citations. Another 3766 citations were excluded based on the title or abstract. The literature was almost entirely identified and retrieved from electronic bibliographic sources. No studies were identified from hand searching reference lists provided in the studies that met the inclusion criteria. A total of 171 studies were assessed for inclusion using a review of the full text. A total of 133 studies were excluded at the full text review stage. A total of 38 studies met the inclusion criteria for this review. A flow diagram for inclusion of studies in the systematic review is provided in Figure 1.

### 3.2. Experimental Studies Evaluating Survival

A total of 26 experimental studies evaluated the use of ILE following acute toxicant exposure and included survival as an outcome of interest. Table 1 provides key study characteristics of these studies, while Table S2 summarizes the results from these experimental studies. The studies originated from a geographically diverse group of investigators, including from the United States (*n* = 7), Iran (*n* = 5), New Zealand (*n* = 3), Turkey (*n* = 3), Canada (*n* = 2), Finland (*n* = 2), Australia (*n* = 1), China (*n* = 1), Egypt (*n* = 1), and Israel (*n* = 1). Four animal species, including rats (*n* = 12 studies), pigs (*n* = 7), rabbits (*n* = 6), and dogs (*n* = 1), were used in these studies. The toxicants examined represented several major pharmacologic and pesticide classes, including tricyclic antidepressants (clomipramine, amitriptyline, desipramine; *n* = 10); organophosphate pesticides (malathion, parathion, dichlorvos, diazinon; *n* = 4); calcium channel blockers (verapamil, amlodipine; *n* =3); antipsychotics (haloperidol, clozapine; *n* = 2); other antidepressants (venlafaxine, bupropion; *n* = 2); psychostimulants (methamphetamine); opioid analgesics (tramadol); antihistamines (diphenhydramine), a centrally acting α2-adrenergic agonist (clonidine); and a β-adrenergic blocker (propranolol). Most studies used anesthetized animal models, and several incorporated adjunctive therapies such as atropine, pralidoxime, bicarbonate, cardiopulmonary resuscitation, or vasopressor support, reflecting attempts to model severe clinical poisoning scenarios.

The duration of monitoring following toxicant exposure and ILE administration varied substantially, ranging from 15 min to 48 h. Short-term monitoring periods of 15–120 min predominated in studies involving rapidly acting cardiovascular drugs or antidepressants, whereas organophosphate and centrally acting toxicants were more commonly evaluated over 24–48 h. Across studies, outcomes were mixed. Several investigations demonstrated improved survival with ILE administration, particularly in models involving clomipramine, amlodipine, parathion, diazinon, and dichlorvos poisoning, while others showed no statistically significant benefit or, in a few cases, worse outcomes with ILE treatment. Tricyclic antidepressants were the most extensively studied toxicant class, although findings remained inconsistent across species and experimental designs.

**Figure 2:**
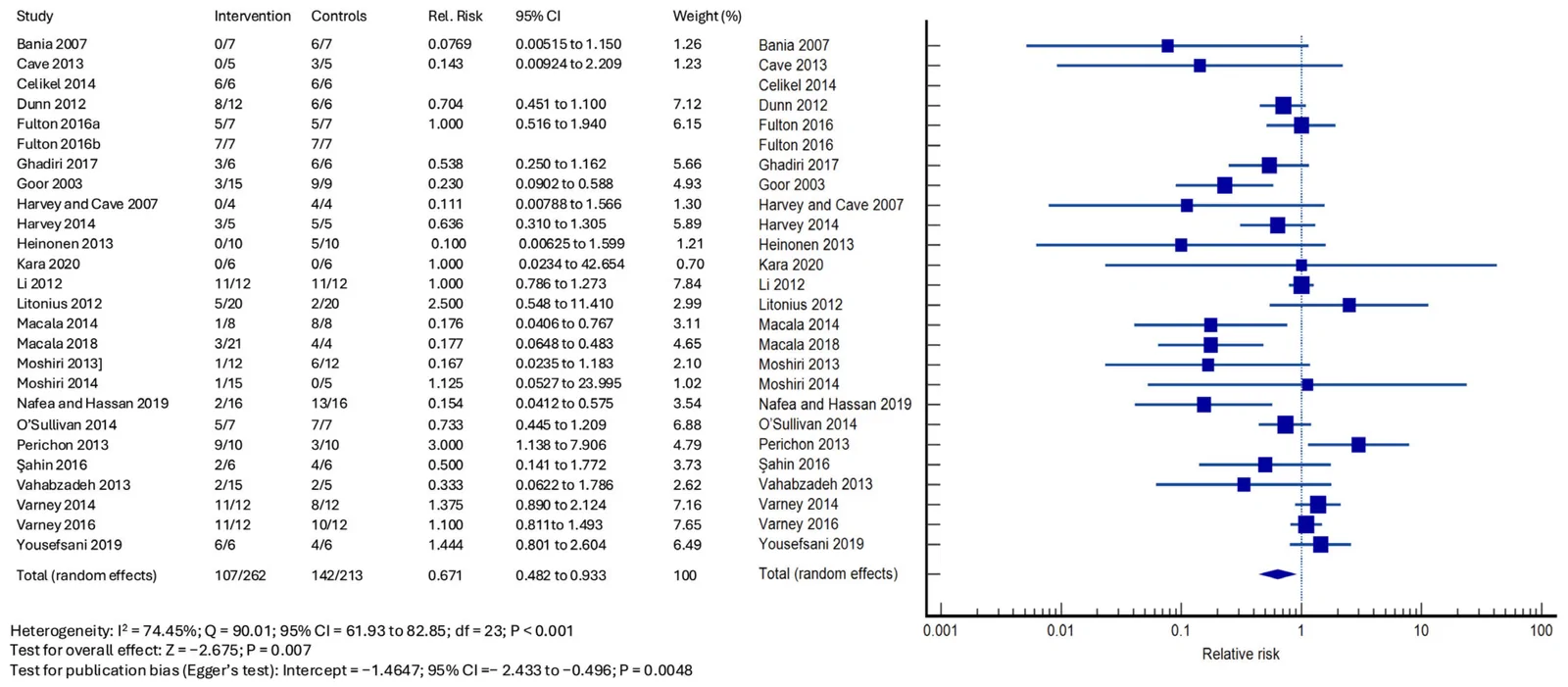
Forest plot analysis of the overall incidence of death in ILE-treated animals following toxicant exposure (global analysis) [26,27,28,29,30,31,32,33,34,35,36,37,38,39,40,41,42,43,44,45,46,47,48,49,50,51].

Figure 2 provides the results of a global (all mortality studies) meta-analysis of the relative risk (RR) of mortality following toxicant exposure followed by ILE administration. Two studies [28,31] reported identical survival proportions in the ILE-treated and control groups (i.e., all animals in both groups either survived or died). These studies remained included in the systematic review but did not contribute an estimable relative risk to the pooled mortality analysis. Consequently, 24 studies contributed to calculation of the pooled effect estimate. Overall, 107/262 (40.8%) animals treated with ILE died, while 142/213 (66.7%) controls died. A pooled estimate of the RR of death following ILE treatment was 0.67 (95% confidence interval: 0.48 to 0.93; *z* = −2.7; *p* = 0.007). Egger’s test indicated significant funnel plot asymmetry/small study effects, raising concern for publication bias (*p* = 0.005). Between-trial heterogeneity was high when using a random effects model (74.5%, 95% CI: 61.9 to 82.9; *p* < 0.001).

The analysis was subsequently broken down for tricyclic antidepressants and organophosphate pesticides to potentially account for the biologic heterogeneity present in the trials (Figure S1). For the ten studies [27,33,34,35,36,37,39,45,46,50] evaluating survival following tricyclic antidepressant exposure and ILE treatment, overall, 36/99 (36.4%) animals treated with ILE died, while 46/88 (52.3%) controls died. A pooled estimate of the RR of death following ILE treatment was 0.75 (95% confidence interval: 0.41 to 1.37; *z* = −1.84; *p* = 0.07). Egger’s test revealed no significant funnel plot asymmetry/small study effects. Between-trial heterogeneity was high when using a random effects model (69.3%, 95% CI: 41.0 to 84.1; *p* = 0.006).

For the four studies [28,29,38,42] evaluating survival following organophosphate insecticide exposure and ILE treatment, overall, 26/42 (61.9%) animals treated with ILE died, while 29/36 (80.6%) controls died (Figure S2). One study [28] reported identical mortality in the ILE-treated and control groups and therefore did not contribute an estimable effect to the pooled analysis; consequently, three studies contributed to the pooled effect estimate. A pooled estimate of the RR of death following organophosphate insecticide exposure and ILE treatment was 0.72 (95% confidence interval: 0.35 to 1.45; *z* = −0.92; *p* = 0.36). Egger’s test revealed no significant funnel plot asymmetry/small study effects. Between-trial heterogeneity was high when using a random effects model (80.3%, 95% CI: 37.7 to 93.7; *p* = 0.006).

Nineteen studies [26,27,28,29,32,33,34,35,37,38,40,41,42,43,44,46,47,48,51] evaluating survival following toxicant exposure and ILE treatment in non-swine animal models were included in a second meta-analysis. One study [28] reported identical mortality in the ILE-treated and control groups and therefore did not contribute an estimable effect to the pooled analysis; consequently, 18 studies contributed to the pooled effect estimate. Overall, 66/207 (31.9%) animals treated with ILE died, while 102/158 (64.6%) controls died (Figure S3). A pooled estimate of the RR of death following toxicant exposure and ILE treatment in rats, rabbits, and dogs was 0.52 (95% confidence interval: 0.33 to 0.82; *z* = −2.81; *p* = 0.005). Egger’s test revealed significant funnel plot asymmetry/small study effects, raising concern for publication bias (*p* = 0.02). Between-trial heterogeneity was high when using a random effects model (75.9%, 95% CI: 62.4 to 84.5; *p* < 0.0001).

**Figure 3:**
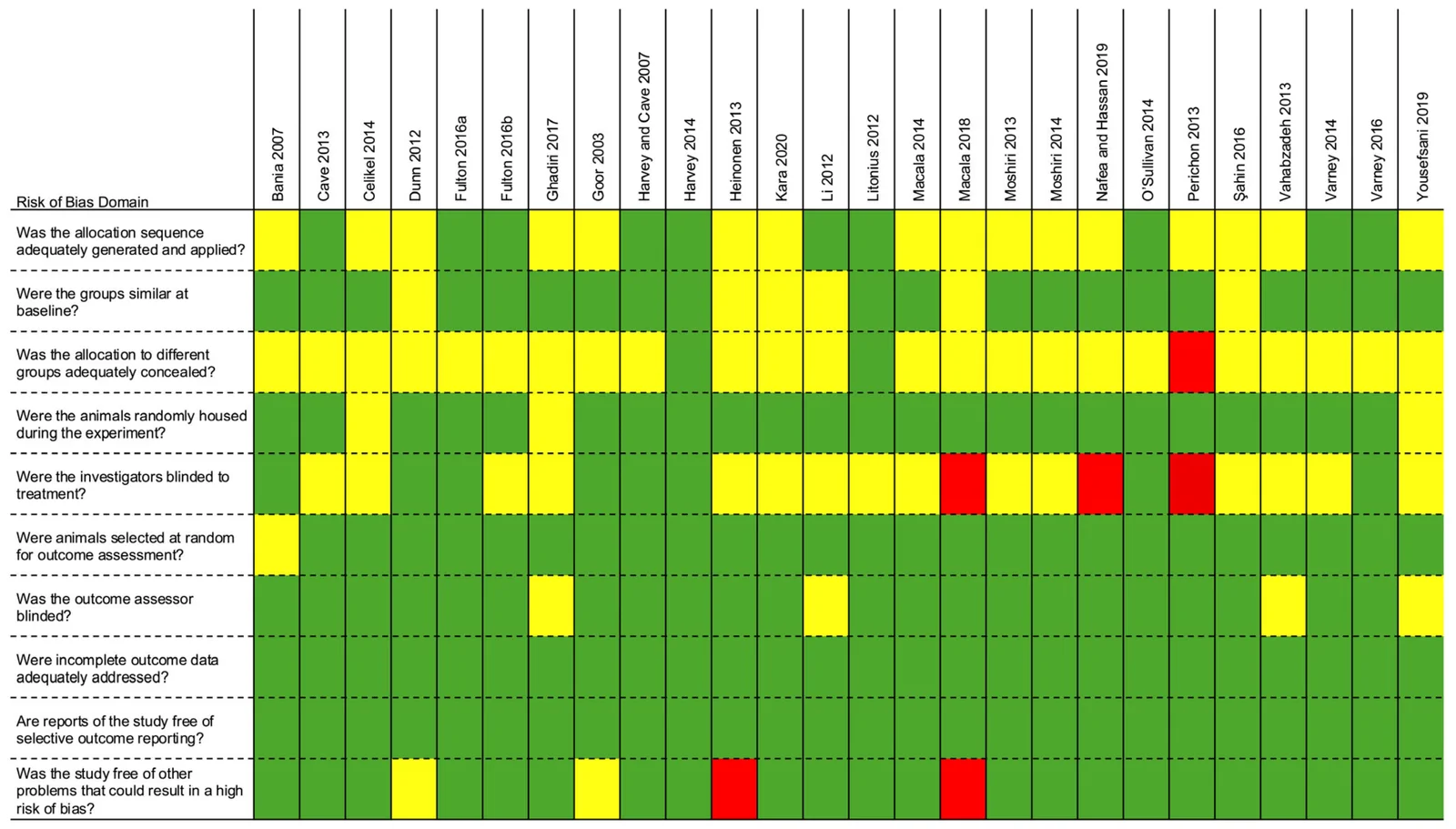
Risk of bias assessment for the 26 included studies evaluating mortality following ILE administration. Key: green: low risk of bias; yellow: unclear risk of bias; red: high risk of bias [26,27,28,29,30,31,32,33,34,35,36,37,38,39,40,41,42,43,44,45,46,47,48,49,50,51].

Summary risk of bias assessments for the included studies are presented in Figure 3. Overall risk of bias for individual criteria is mostly low, but the risk of bias remained unclear for some criteria in some studies. Allocation concealment and blinding of outcome assessment were incompletely reported or unreported in some studies. Blinding of outcome personnel and participants and incomplete outcome data were generally well-reported, and minimal risk of bias associated with these criteria was detected. Risk of bias was mostly low in random sequence generation and in other sources of bias, but, in some studies, the risk of bias for these criteria was unclear.

Overall evidence from the experimental studies evaluating mortality following toxicant exposure and ILE administration was evaluated using GRADE. Using the GRADE framework, the overall certainty of evidence for mortality outcomes in animal models of toxicant poisoning treated with ILE was judged as very low certainty overall (Table 2). Several domains contribute to downgrading confidence in the evidence, including risk of bias, inconsistency, imprecision, and publication bias (Table S3).

### 3.3. Experimental Studies Evaluating Neurological Outcomes

A total of 17 experimental studies evaluated whether ILE use following acute toxicant exposure would improve neurological outcomes. Table 3 provides key study characteristics of these studies, while Table S4 summarizes these experimental studies. Studies originated predominantly from Iran (*n* = 5) and Turkey (*n* = 5), followed by Brazil (*n* = 2) and the United States (*n* = 2), with single studies from Germany, Japan, and China. The one Japanese study evaluated two anesthetic toxicants (sevoflurane and isoflurane). Rats were the most used species (*n* = 11 studies), followed by rabbits (*n* = 4), while mouse and turtle models were each represented in one study. Toxicants included organophosphate pesticides and cholinesterase inhibitors (malathion [*n* = 2], methyl parathion, chlorpyriphos, diazinon, dichlorvos; *n* = 6 studies); sedative–hypnotic or anesthetic agents were evaluated in three studies (diazepam, pentobarbital, and sevoflurane/isoflurane), with the latter two agents evaluated within a single study (*n* = 3); antipsychotics (haloperidol, clozapine; *n* = 2), recreational or stimulant drugs (delta-9-tetrahydrocannabinol, methamphetamine; *n* = 2), antiparasitic agents (ivermectin; *n* = 1), opioids (tramadol; *n* = 1), brevetoxin (*n* = 1), and 2-glyphosate isopropylamine herbicide (*n* = 1) were also evaluated. Time from toxicant exposure to ILE administration varied considerably, ranging from less than 30 s to 12 h, although most studies administered ILE within 30 min of exposure.

Neurological outcomes assessed included clinical neurological observations, seizure incidence and severity, locomotor activity, righting reflex recovery, rotarod performance, anesthesia duration, histopathology, and brain immunohistochemistry. Several studies reported potential neurological benefits associated with ILE administration. Improvements were observed in methamphetamine-induced behavioral abnormalities and neuronal apoptosis, brevetoxin-associated paralysis and ataxia, tramadol-induced seizures, clozapine-associated seizure scores and motor performance, and recovery from inhaled anesthetic exposure. Histopathologic or immunohistochemical evidence suggesting reduced neuronal injury was also reported in selected studies involving glyphosate, chlorpyriphos, methamphetamine, and methyl parathion exposure. However, findings were inconsistent across toxicants and outcome measures. Multiple studies demonstrated no statistically significant neurological improvement with ILE, including investigations involving diazepam, ivermectin, malathion, and dichlorvos. In some cases, neurological outcomes worsened following ILE administration, such as increased cataleptic scores in haloperidol-treated rabbits.

**Figure 4:**
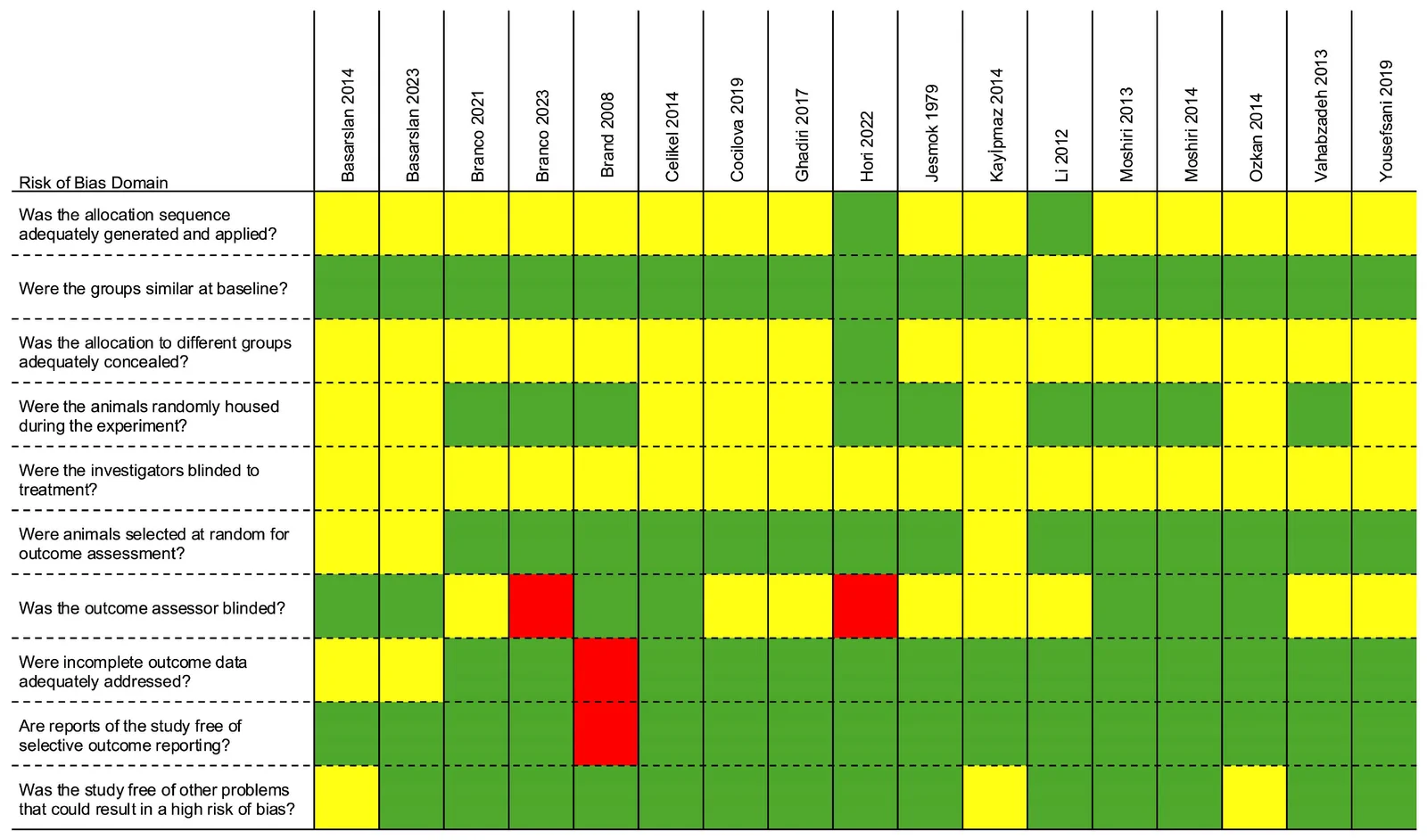
Risk of bias assessment for the 17 included studies evaluating neurological outcomes. Key: green: low risk of bias; yellow: unclear risk of bias; red: high risk of bias [28,32,38,42,43,48,51,52,53,54,55,56,57,58,59,60,61].

Summary risk of bias assessments for the included studies are presented in Figure 4. Overall risk of bias for individual criteria is moderate, as the risk of bias remained unclear for multiple criteria in some studies. Allocation, random housing, and blinding were unreported in most studies. Blinding was considered a critical risk of bias for studies evaluating behavior, clinical signs, and other neurological outcomes because these outcomes frequently relied on subjective assessment. In such studies, knowledge of treatment assignment could influence outcome assessment and potentially bias estimated treatment effects in favor of or against ILE. Bias associated with differences in groups at baseline, random selection of animals for outcome assessment, incomplete outcome data, selective outcome reporting, and other sources of bias was generally low in most studies.

Overall evidence from the experimental studies evaluating neurological outcomes following toxicant exposure and ILE administration was evaluated using GRADE. Using the GRADE framework, the overall certainty of evidence for neuropathology, neurological signs, and behavior in animal models of toxicant poisoning treated with ILE was judged as very low certainty overall (Table 4). The overall certainty of evidence for improved recovery from anesthesia in animal models of toxicant poisoning treated with ILE was judged as low certainty overall (Table 4). Several domains contribute to downgrading confidence in the evidence, including risk of bias, inconsistency, imprecision, and publication bias (Table S5).

### 3.4. Randomized Clinical Trials

Table 5 summarizes two randomized clinical trials evaluating ILE therapy in naturally occurring veterinary toxicant exposures. The studies originated from Sweden (*n* = 1) and Australia (*n* = 1), and both utilized feline clinical populations. The Swedish study [62] was a prospective randomized clinical study that evaluated cats intoxicated with alpha-chloralose. Although all animals survived and recovered within 72 h, no statistically significant differences were identified between ILE-treated and control groups for serum alpha-chloralose concentrations or intoxication scores. The Australian randomized clinical trial [63] evaluated cats with permethrin toxicosis treated with ILE versus saline. The investigators reported earlier improvement in clinical stage among ILE-treated cats compared with controls (*p* < 0.05), suggesting potential clinical benefit.

**Figure 5:**
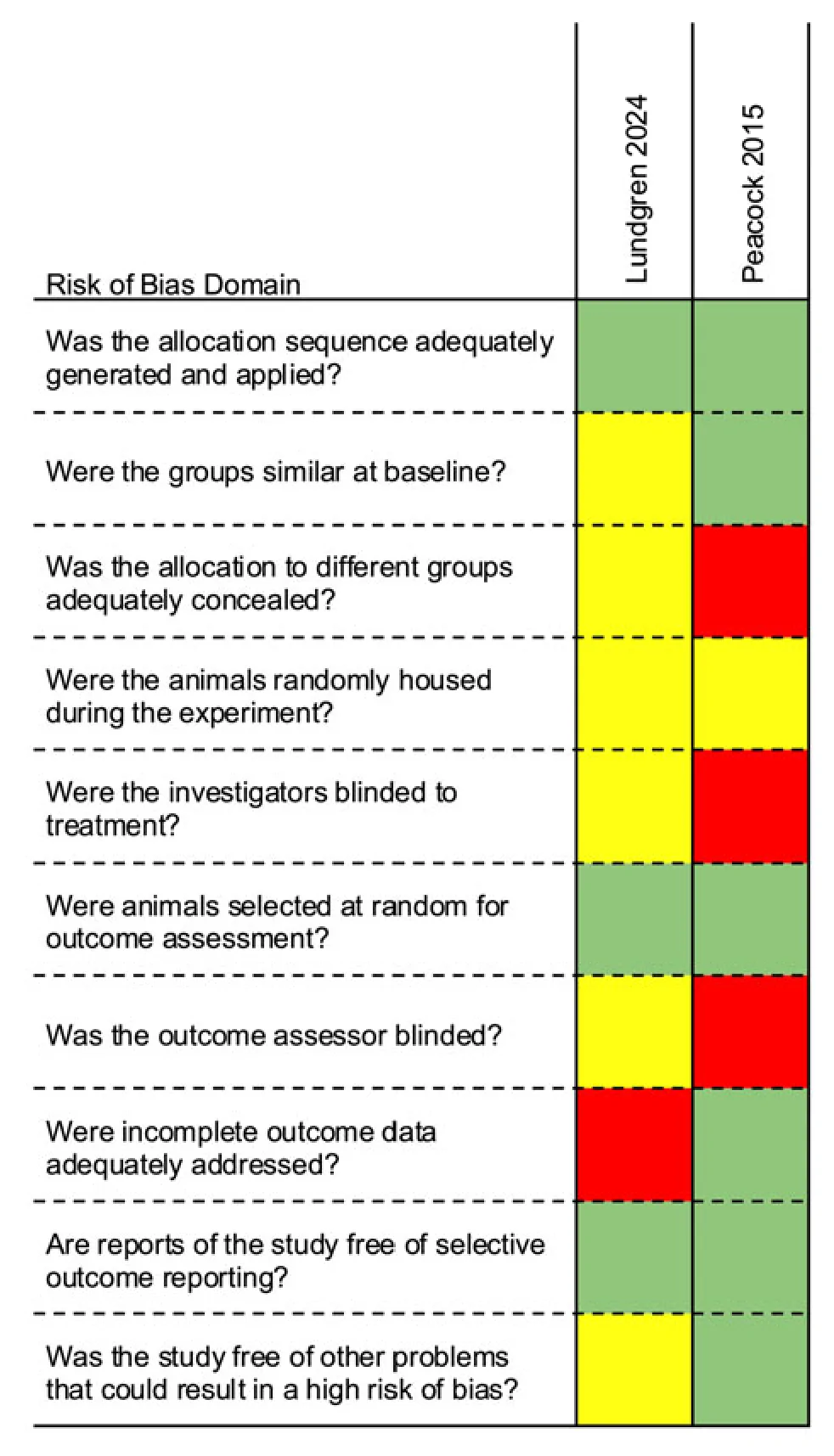
Risk of bias assessment for the two included RCTs. Key: green: low risk of bias; yellow: unclear risk of bias; red: high risk of bias [62,63].

Summary risk of bias assessments for the two included RCTs are presented in Figure 5. Overall risk of bias for individual criteria is high, as the risk of bias remained unclear for multiple criteria in both studies. Risk of bias was deemed high in one of the two studies for allocation, blinding, and incomplete reporting of outcomes. Insufficient reporting hampered assessment of bias associated with baseline characteristics, housing, and other sources of bias. Blinding was considered a critical risk of bias, as evaluation of clinical signs and other subjective neurological outcomes could be affected if individuals have knowledge of treatment effects. Because both RCTs evaluated neurological signs, evidence from these studies was considered in the GRADE assessment for this outcome (Table 4).

## 4. Discussion

This systematic review analyzes the impact of ILE treatment on poisoned animals’ survival and neurological recovery. The search included peer-reviewed electronic and print resources with no restrictions on language or publication date. We found that reporting of the methodological features of studies was often incomplete, making evaluation of risk of bias within and across studies difficult. Incomplete reporting of methodological details in experimental animal studies and animal-centric systematic reviews has been noted by others [64,65].

### 4.1. Survival Following ILE Treatment of Poisoned Animals

Our systematic review found a diverse range of experiments that varied by species, toxicants, ILE dosages and timing of administration, assessment durations, and outcomes of interest, among other factors. When considered collectively in our global meta-analysis, ILE was associated with lower mortality in toxicant-poisoned animals (RR = 0.67; 95% confidence interval: 0.48 to 0.93; *p* = 0.007). However, high heterogeneity, possible publication bias, imprecision, and methodological limitations resulted in very low certainty in the body of evidence and limit the generalizability of the pooled estimate. Although the pooled analysis demonstrated lower mortality with ILE, the magnitude and generalizability of this association across toxicants and species remain uncertain.

Because previous meta-analytic work reported species-related differences in ILE treatment effects and proposed that swine may respond differently to lipid emulsion [66,67], we performed an exploratory sensitivity analysis excluding swine studies. This analysis was intended to assess the influence of swine studies on the pooled estimate rather than assuming a priori that swine constitute an invalid experimental model. We subsequently evaluated 19 studies that examined survival following toxicant exposure and ILE treatment in a non-swine animal model, and 18 studies contributed an estimable effect to the pooled analysis. A pooled estimate of the relative risk of death following toxicant exposure and ILE treatment in rats, rabbits, and dogs was 0.52 (95% confidence interval: 0.33 to 0.82; Z = −2.81; *p* = 0.005). Although the pooled estimate was statistically significant after exclusion of swine studies, high heterogeneity and possible publication bias substantially limit interpretation of this finding. Results from our meta-analysis that excluded pigs are qualitatively similar to those reported by others [66]. Fettiplace and Pichurko [66] reported that ILE treatment of animals other than pigs reduced the chance of death with an odds ratio of death of 0.26. This prior systematic review included exposures to local anesthetic agents and, in some cases, treatment with ILE occurred prior to toxicant exposure [66]. These results have prompted some scientists to advise against the use of swine as an animal model for evaluating the efficacy of ILE as a treatment for toxicant exposure [67]; however, the present analysis does not establish that swine are intrinsically unsuitable models or that species differences account for the observed subgroup findings.

The toxicant-specific analyses of tricyclic antidepressant and organophosphate insecticide studies did not demonstrate statistically significant reductions in mortality with ILE. This is in contrast with recent systematic reviews and meta-analyses that found that ILE lowered the mortality rate in human patients poisoned with organophosphate pesticides [68,69]. The reasons for this apparent difference are uncertain. The experimental animal studies and human clinical studies differ substantially in study design, species, pesticide exposure conditions, supportive care, timing of ILE administration, and outcome assessment, limiting direct comparison between these bodies of evidence. In particular, the experimental animal studies may not reflect naturally occurring clinical exposures. Consequently, the apparent difference in treatment effect for organophosphate pesticides should not necessarily be interpreted as evidence of a species-specific difference in the efficacy of ILE. The organophosphate-specific pooled estimate was imprecise, with only three studies contributing an estimable effect and a wide confidence interval spanning substantial benefit and no treatment effect.

Our global meta-analysis identified high heterogeneity when using a random effects model. The substantial clinical and methodological heterogeneity among studies likely contributed to the observed statistical heterogeneity. Potential sources included animal species, toxicant class, toxicant dose and route, ILE dose and route, timing of ILE administration, use of anesthesia and supportive interventions, and duration of follow-up. However, the small number of studies within most combinations of these characteristics precluded reliable meta-regression or sufficiently powered subgroup analyses to determine the independent contribution of these factors.

Our systematic review and meta-analysis were used to inform the application of the GRADE framework. Our analysis found that the current experimental evidence provides insufficient certainty to conclude that ILE consistently reduces mortality in animals following toxicant exposure. Although some studies demonstrate increased survival, the evidence base is limited by small sample sizes, substantial risk of bias, heterogeneity, and imprecision. The reviewed studies varied considerably across toxicants, animal species, timing of ILE administration, and co-interventions. Many animals were anesthetized and mechanically supported, conditions that differ substantially from veterinary practice. Anesthesia and mechanical support could reduce manifestations such as seizures or respiratory compromise and thereby attenuate an observable treatment effect while simultaneously altering toxicant disposition, cardiovascular physiology, or treatment response. Toxicant doses were often supralethal or administered intravenously rather than through clinically relevant exposure routes. Monitoring periods were also short in several studies (15–120 min), limiting assessment of delayed mortality. The overall body of evidence suggests that ILE may have benefits in selected toxicant exposures; however, the true magnitude and consistency of effect remain highly uncertain. Although the pooled analysis demonstrated lower mortality with ILE, substantial between-study heterogeneity and very low certainty mean that the magnitude and applicability of this association across species and toxicants remain uncertain. Clinical decisions regarding ILE should therefore consider the specific toxicant, severity of poisoning, availability of established toxicant-specific and supportive therapies, and the uncertainty of the evidence supporting ILE.

### 4.2. Neurological Outcomes in Poisoned Animals Following ILE Treatment

A second objective of our review was to evaluate whether ILE treatment reduces the incidence or severity of neurological effects in poisoned animals. A total of 17 experimental animal studies met our inclusion criteria. We performed a qualitative analysis of studies across four outcome domains: neuropathology, neurological signs, behavior, and recovery from anesthesia. A qualitative approach was used because quantitative measures suitable for metanalytic approaches were unavailable in most studies. Eight animal studies evaluated neuropathologic outcomes following toxicant exposure and ILE treatment. Studies included rats and rabbits exposed to organophosphate pesticides (malathion, methyl parathion, chlorpyriphos), glyphosate isopropylamine, methamphetamine, ivermectin, and haloperidol [28,32,43,52,53,54,60,61]. Neuropathologic outcomes included histopathology scores, apoptotic markers (caspase-3, TUNEL), ultrastructural blood–brain barrier changes, and neurodegeneration scores. Follow-up periods ranged from 10 min to 3 days after ILE administration. Across studies, several investigations reported reductions in apoptotic markers or histologic injury following ILE administration, including decreases in caspase-3-positive cells, TUNEL-positive cells, apoptotic neurons, or blood–brain barrier injury. However, findings were inconsistent, quantitative data were frequently incomplete, and several studies demonstrated no statistically significant differences in neuropathologic endpoints. Using the GRADE framework, the certainty of evidence supporting improvement in neuropathologic outcomes with ILE therapy in animal poisoning models was rated as very low.

Ten experimental animal studies evaluated neurological or clinical neurological signs [28,38,42,43,48,51,54,55,57,60]. Species included rats, rabbits, and pond sliders exposed to ivermectin, diazepam, malathion, brevetoxin, methyl parathion, dichlorvos, diazinon, haloperidol, tramadol, and clozapine. Outcomes included neurological examination scores, seizure incidence and duration, tremor intensity, paralysis, fasciculations, muscle tone abnormalities, catalepsy, ataxia, lethargy, and other observable neurological signs. Several studies demonstrated improvement in neurological manifestations following ILE administration, particularly for brevetoxin, haloperidol, tramadol, and clozapine exposure [43,48,51,57]. Reported benefits included reductions in seizure incidence or duration, improved ambulation, decreased lethargy, and reduced severity of cataleptic or neurological signs. A recent narrative review of the human literature suggests that ILE treatment decreases coma scores caused by toxic doses of amitriptyline, trazodone, and other lipid-soluble neuropsychiatric drugs [5]. However, many experimental studies evaluated in our review reported no statistically significant differences between treatment groups, and outcome measures were heterogeneous and frequently subjective.

There were also two randomized clinical trials that evaluated the use of ILE in poisoned animals [62,63]. An Australian randomized clinical trial involved 34 treatment-naïve cats exposed to permethrin. Cats treated with ILE had milder neurological signs when compared with controls [63]. The second study was a prospective, randomized clinical study that evaluated the efficacy of ILE in cats exposed to alpha-chloralose [62]. All cats in this study survived and there was no significant difference in the severity of the neurological signs observed following alpha-chloralose exposure.

Three experimental animal studies [32,51,56] evaluated behavioral outcomes following toxicant exposure and treatment with ILE. Species included mice and rats exposed to THC, methamphetamine, and clozapine. Behavioral outcomes included analgesic response (tail-flick latency), locomotor activity, stereotyped behaviors, freezing response, rearing behavior, and motor coordination assessed by rotarod performance. Findings were mixed across these three studies. One study demonstrated no effect of ILE on THC-induced analgesia [56], whereas two studies reported improvement in selected behavioral outcomes following ILE administration, including increased locomotor and rearing activity, reduced stereotyped behaviors, and improved rotarod performance in animals given either methamphetamine or clozapine and treated with ILE [32,51]. However, behavioral effects were variable across time points and endpoints, and evidence was limited by small sample sizes and heterogeneous assessment methods. The overall certainty of evidence supporting improvement in behavioral outcomes with ILE treatment in animal toxicology studies is very low according to the GRADE framework.

Two experimental animal studies [58,59] evaluated recovery from anesthesia or sedation following administration of ILE. Studies were conducted in rats exposed to sevoflurane, isoflurane, or pentobarbital. Outcomes included time to restoration of righting reflex and duration of sleep or anesthetic effect. Follow-up periods ranged from minutes to several hours and primarily assessed acute recovery endpoints. Unlike other neurological outcome categories, findings across these studies were relatively consistent and demonstrated uniform improvement in anesthetic recovery endpoints following ILE administration. The overall certainty of evidence supporting accelerated recovery from anesthesia or sedation with ILE treatment in animal studies is low according to the GRADE framework. Confidence in the evidence remains limited by small sample sizes, incomplete methodological reporting, and uncertain translational applicability to clinical veterinary toxicology. Among the neurological outcome domains evaluated in this review, accelerated recovery from anesthesia or sedation represented the most consistent positive finding. However, this evidence was derived from only two small experimental studies and was rated as low certainty. Whether the observed effect reflects altered anesthetic distribution, clearance, or another mechanism cannot be determined from the available studies. Moreover, the clinical relevance of these findings to naturally occurring toxicant exposures or routine anesthetic recovery in veterinary patients remains uncertain.

### 4.3. Study Limitations and Next Steps

An important limitation of this review is deviation from the registered protocol. The protocol specified inclusion of the gray literature; however, the completed review was restricted to peer-reviewed publications. Consequently, potentially relevant unpublished studies, dissertations, and conference proceedings may have been omitted that may influence the magnitude of the pooled treatment effect. Our review also excluded case reports and case series. This decision reflects our experience conducting a prior scoping review of the animal clinical literature that found that in most case reports and case series ILE was used in conjunction with other treatments [19]. Co-administration of other drugs can confound the ability to conclude whether clinical improvements reported following ILE administration could be directly attributed to the use of ILE. Case reports and case series also lack an appropriate comparator group. Sex was also incompletely represented across the experimental literature, with many studies using only male animals. Although sex differences in lipid metabolism and toxicokinetics have been reported, the studies included in this review were not designed to determine whether sex modifies the efficacy of ILE following toxicant exposure. Future studies incorporating both sexes and adequately powered analyses are needed to determine whether sex influences the therapeutic response to ILE. Another limitation of our review relates to our focus on neurotoxicoses. This focus reflected the findings of our scoping review which found that most of the clinical case reports and case series involved animals exposed to a suspected neurotoxicant or animals demonstrating neurological effects at presentation [19]. One challenge in our systematic review was defining a neurotoxicant. Rather than rely on a narrow definition we sought experimental animal studies where survival or one or more neurological outcome was evaluated. An additional limitation is that, although the registered protocol anticipated quantitative synthesis when appropriate, the specific meta-analytic model and subgroup analyses were not prespecified. These analyses were developed after examination of the eligible evidence and should therefore be regarded as exploratory. Consequently, these analyses may be more susceptible to data-dependent analytical decisions than analyses specified prospectively. One final limitation is that we excluded human studies.

## 5. Conclusions

An exploratory meta-analysis demonstrated lower mortality in ILE-treated animals following non-local anesthetic toxicant exposure. A sensitivity analysis excluding swine studies also demonstrated lower mortality, with a lower pooled relative-risk point estimate than in the global analysis. However, substantial between-study heterogeneity, possible publication bias, methodological limitations, and very low certainty of evidence limit confidence in the magnitude and generalizability of these pooled estimates. Neurological outcomes were heterogeneous; accelerated recovery from anesthesia or sedation was the most consistent positive neurological finding but was supported by only two small experimental studies and low-certainty evidence.

The overall certainty of evidence was very low for mortality and most neurological outcomes and low for recovery from anesthesia. Accordingly, the available evidence does not support routine use of ILE as a standard treatment for non-local anesthetic poisoning in animals; its use should instead be considered on a case-by-case basis in conjunction with established supportive and toxicant-specific therapies.

Interpretation and clinical translation of these findings are limited by small sample sizes, high heterogeneity among toxicants and experimental models, incomplete methodological reporting and risk of bias, potential publication bias, and the limited number of controlled clinical trials in veterinary patients. In addition, many experimental models differed substantially from naturally occurring poisoning with respect to toxicant dose and route, supportive care, and duration of follow-up. Future studies should prioritize adequately powered, randomized and blinded studies of specific toxicant–species combinations using clinically relevant exposure models, standardized neurological and survival outcomes, and sufficient follow-up to detect clinically meaningful treatment effects. High-quality controlled clinical trials in naturally poisoned veterinary patients are particularly needed to determine which toxicants and clinical circumstances, if any, are most likely to benefit from ILE therapy.

## Figures and Tables

**Table 1:** Overview of experimental studies evaluating survival.

Study	Country of Publication	Species/Breed or Strain/Age/Sex	Toxicant	Toxicant Dose (Route)	Groups	Lipid Emulsion Dose, Route (Source)	Time Since Exposure for ILE Administration
Bania 2007 [26]	United States	Dog/NR/adult/M	Verapamil	6 mg/kg/h (IV) until 50% of MAP, then additional 0.5 h prior to ILE, then 2 mg/kg/h (IV) for a total 2 h	ILE (*n* = 7); control (*n* = 7; saline)	Bolus: 7 mL/kg, 30 min, IV (20% IFE Sigma-Aldrich)	45 min
Cave 2013 [27]	New Zealand	Rabbit/NZW/adult/M and F	Clomipramine	3.2 mg/kg/min to 50% baseline MAP and then at 1.6 mg/kg/min for 30 min (or death); total mean clomipramine dose to death or 30 min = 25.7 and 59.3 mg/kg for bicarbonate and ILE, respectively	ILE (*n* = 5), 3 mL/kg 8.4% bicarbonate (*n* = 5)	Bolus: 3 mL/kg Intralipid 20%, 60 s, IV (NR)	<1 min
Celikel 2014 [28]	Turkey	Rat/Wistar/adult/M	Malathion	1150 mg/kg (PO)	ILE (*n* = 6); control (*n* = 6; NR)	12.4 mL/kg, IP (NR)	5 min
Dunn 2012 [29]	United States	Rat/Wistar/NR/M	Parathion	54 mg/kg (PO)	ILE 5 min delay (*n* = 6); ILE 20 min delay (*n* = 6); control (*n* = 6; saline)	Bolus: 15 mL/kg, 3 min every 20 min, IV (20% IFE; Fresenius Kabi)	5 or 20 min
Fulton 2016a [30]	United States	Pig/Yorkshire/55 to 70 kg/M	Venlafaxine	40 to 50 mg/kg (IV)	ILE (*n* = 7); control (*n* = 7; CPR alone)	NR	2 min
Fulton 2016b [31]	United States	Pig/Yorkshire/55 to 76 kg/M	Bupropion	35 mg/kg (IV)	ILE (*n* = 7); control (*n* = 7; CPR alone)	Bolus: 4 mL/kg, 2 min; CRI: 0.5 mL/kg/min, 10 min, IV (NR)	2 min
Ghadiri 2017 [32]	Iran	Rat/Wistar/adult/M	Methamphetamine	45 mg/kg (IP)	ILE (*n* = 6); control (*n* = 6; saline)	Bolus: 18.6 mL/kg Intralipid 20%, IV (Fresenius Kabi)	5–7 min
Goor 2003 [33]	Israel	Rat/Wistar/adult/M	Clomipramine	12.5 mg (IV)	ILE (*n* = 12); control (*n* = 9; saline)	Bolus: 2.5 mL Intralipid 10%, IV (Pharmacia)	0 min
Harvey and Cave 2007 [34]	New Zealand	Rabbit/NZW/adult/M and F	Clomipramine	8 mg/ml at 40 mL/h (IV) until 50% baseline MAP; total mean clomipramine dose = 9.2 and 11.1 mg/kg for control and ILE, respectively (NSD)	ILE (*n* = 10); control (*n* = 10; saline)	Bolus: 12 mL/kg Intralipid 20%, 4 min, IV (NR)	<1 min
Harvey 2014 [35]	New Zealand	Rabbit/NR/adult/M and F	Clomipramine	3.2 mg/kg/min (IV) until 50% baseline MAP, then reduced to 2 mg/kg/min; total mean clomipramine dose at 50% reduction in MAP = 7.4 (control), 11.9 (ILE), and 7.6 (ILE + plasma exchange groups), respectively (NSD)	ILE (*n* = 5); ILE followed by plasma exchange (*n* = 5); control (*n* = 5; saline); sodium bicarbonate (*n* = 5)	Bolus: 1.5 mL/kg IV; CRI: 15 mL/kg/h; Intralipid 20%, IV (Pharmatel Fresenius Kabi)	<1 min
Heinonen 2013 [36]	Finland	Pig/Landrace/NR/M and F	Amitriptyline	10 mg/kg (IV)	ILE (*n* = 10); control (*n* = 10; Ringers)	Bolus: 1.5 mL/kg, 1 min; CRI: 0.25 mL/kg/min for 29 min, IV (Baxter SA 20% lipid emulsion, ClinOleic)	30 min
Kara 2020 [37]	Turkey	Rabbit/NZW/NR/M and F	Clomipramine	70 mg/kg (PO) until MAP reached 55–60% of baseline	ILE (*n* = 6/dose group); control (*n* = 6)	Bolus: 1.5 mL/kg, NR; CRI: 0.25 mL/kg/min for up to 2 h, IV (NR)	30–45 min
Li 2012 [38]	China	Rat/Sprague Dawley/adult/M and F	Dichlorvos	11 mg/kg (IP)	ILE (*n* = 12/dose group); control (*n* = 12; saline)	Bolus: 5 mL/kg, NR, 20% fat emulsion, IV (Shandong Huarui Pharmaceutical)	<0.5 min
Litonius 2012 [39]	Finland	Pig/Landrace/NR/M and F	Amitriptyline	15 mg/kg (IV)	ILE (*n* = 10); control (*n* = 10; Ringers)	Bolus: 1.5 mL/kg, 1 min; CRI: 0.25 mL/kg/min for 29 min 20% lipid emulsion, IV (Baxter SA, ClinOleic)	<0.5 min
Macala 2014 [40]	Canada	Rat/Sprague Dawley/adult/M	Clonidine	2.5 μg/kg/min for 60 min (IV)	ILE (*n* = 8); control (*n* = 8; saline)	Bolus: 20% solution, 1.0 mL/kg (NR)	30 min
Macala 2018 [41]	Canada	Rat/Sprague Dawley/adult/M	Propranolol	15 mg/kg (IV)	ILE (*n* = 21); control (*n* = 4; saline)	Bolus: 20% solution, 1.0, 2.0, or 3.0 mL/kg (Fresenius Kabi AB)	<0.5 min
Moshiri 2013 [42]	Iran	Rat/Sprague Dawley/adult/M	Diazinon	480 mg/kg (PO)	ILE 10% (*n* = 6); ILE 20% (*n* = 6); saline 16 mL/kg (*n* = 6); saline 10 mL/kg (*n* = 6)	Bolus: 10 mL/kg,15 min, ILE 10% or ILE 20%, IV (Fresenius Kabi)	30 min
Moshiri 2014 [43]	Iran	Rabbit/NZW/adult/M	Haloperidol (HAL)	2.6 mg/kg (IV)	Water + saline (*n* = 5); water + ILE 18 (*n* = 5); HAL + saline (*n* = 5); HAL + ILE 6, 12, or 18 (*n* = 15)	Bolus: 6, 12, or 18 mL/kg Intralipid 20%, IV (Fresenius Kabi)	30 min
Nafea and Hassan 2019 [44]	Egypt	Rat/NR-albino/adult/M	Amlodipine	42 mg/kg (IP)	ILE (*n* = 16); control (*n* = 16; saline)	Bolus: 24.8 mL/kg, 10 min, at 5, 30, and 60 min SMOFlipidVR 20%, IV (Pharmatel Fresenius Kabi)	<0.5 min
O’Sullivan 2014 [45]	United States	Pig/Yorkshire/55 to 75 kg/M	Desipramine	7–10 mg/kg (IV)	ILE (*n* = 7); control (*n* = 7; CPR alone)	Bolus: 2 mL/kg, 2–3 min; CRI: 0.25 mL/kg/min, 10 min 20% ILE, IV (NR)	2 min
Perichon 2013 [46]	Australia	Rat/Wistar/adult/M	Amitriptyline	70 mg/kg (PO)	ILE (*n* = 10); control (*n* = 10; Hartmann’s solution; 8.4% sodium bicarbonate)	Bolus: 4 mL/kg, 5 min; CRI: 4 mL/kg/h 20% Intralipid, IV (Fresenius Kabi)	30 min
Şahin 2016 [47]	Turkey	Rat/Sprague Dawley/adult/NR	Verapamil	2.5 mg/kg/min (IV) until MAP reached 60% of baseline	ILE (*n* = 6); control (*n* = 6; saline)	Bolus: 12.4 mL/kg, 5 min, 20% Intralipid, IV (Fresenius Kabi)	<0.5 min
Vahabzadeh 2013 [48]	Iran	Rabbit/NZW/adult/M	Tramadol	50 mg/kg (IV)	ILE (*n* = 15, 5/group); control (*n* = 5; saline)	Bolus: 6, 12, or 18 mL/kg ILE 20%, IV (Fresenius Kabi AB)	30 min
Varney 2014 [49]	United States	Pig/NR/45 to 55 kg/F	Amitriptyline	0.5 mg/kg/min (IV) until MAP reached 60% of baseline, then reduced to 0.05 mg/kg/min	ILE (*n* = 12); control (*n* = 12; saline + 8.4% sodium bicarbonate)	Bolus: 7 mL/kg; CRI: 0.25 mL/kg/min, IV (NR)	30 min
Varney 2016 [50]	United States	Pig/Yorkshire/45 to 55 kg/F	Diphenhydramine	1 mg/kg/min (IV) until MAP reached 60% of baseline; total infused: control: 1645 mg; ILE: 1510 mg	ILE (*n* = 12); control (*n* = 12; saline + 8.4% sodium bicarbonate)	Bolus: 7 mL/kg; CRI: 0.25 mL/kg/min, IV (NR)	30 min
Yousefsani 2019 [51]	Iran	Rat/Wistar/93–96 d/M	Clozapine	40 mg/kg (IV)	ILE (*n* = 6); control (*n* = 6; saline)	Bolus: 18.6 mL/kg ILE 20%, IV (Fresenius Kabi AB)	15 min

Abbreviations: CRI: continuous rate infusion; F: female; HAL: haloperidol; IV: intravenous; M: male; MAP: mean arterial pressure; NR: not reported; NSD: no significant difference; NZW: New Zealand white; PO: per os.

**Table 2:** Profile of confidence in the body of evidence on whether in animal models of toxicant poisoning treatment with ILE, compared with no ILE treatment, improves survival. No upgrades occurred. Key: — if minimal or no concern was present; ↓ if serious concern to downgrade confidence by one point. A detailed rationale for judgments regarding risk of bias, indirectness, inconsistency, imprecision, and publication bias is provided in Table S3.

	Initial Confidence Rating	Downgrades					Certainty Rating
Studies		Risk of Bias	Indirectness	Inconsistency	Imprecision	Publication Bias	
26 experimental studies [26,27,28,29,30,31,32,33,34,35,36,37,38,39,40,41,42,43,44,45,46,47,48,49,50,51] Dog (*n* = 1); pig (*n* = 7); rabbit (*n* = 6); rat (*n* = 12)	4	↓	—	↓	↓	↓	Very Low

**Table 3:** Overview of experimental studies evaluating neurological outcomes.

Study	Country of Publication	Species/Breed or Strain/Age/Sex	Toxicant	Toxicant Dose (Route)	Groups	Lipid Emulsion Dose, Route (Source)	Time Since Exposure for ILE Administration
Basarslan 2014 [52]	Turkey	Rat/NR/NR/NR	Malathion	10 mg/kg (PO)	ILE (*n* = 8/time point); control (*n* = 8; NR)	18.6 mL/kg, PO (NR)	0, 6, 12 h
Basarslan 2023 [53]	Turkey	Rat/Wistar/adult/F	2-Glyphosate isopropylamine	4 mg/kg/day (PO)	ILE (*n* = 7); control (*n* = 7; NR)	18.6 mL/kg, PO (NR)	10 min
Branco 2021 [54]	Brazil	Rabbit/NZW/adult/M	Ivermectin	80 mg/kg (PO)	ILE (*n* = 6); control (*n* = 6; saline)	Bolus: 2 mL/kg; CRI: 0.25 mL/kg/min for 60 min, IV (Lipovenos 20%, Fresenius Kabi)	3 h
Branco 2023 [55]	Brazil	Rabbit/NZW/adult/M	Diazepam	1 mg/kg (IV)	ILE (*n* = 6); control (*n* = 6; saline)	Bolus: 1.5 mL/kg; CRI: 0.25 mL/kg/min for 60 min, IV (Lipovenos 20%, Fresenius Kabi)	10 min
Brand 2008 [56]	Germany	Mouse/129S2/SVHsd/adult/M	Delta-9-tretrahydrocannabinol	50 mg/kg (IP)	ILE (*n* = 10); control (*n* = 10; NR)	(NR)	15 min
Celikel 2014 [28]	Turkey	Rat/Wistar/adult/M	Malathion	1150 mg/kg (PO)	ILE (*n* = 6); control (*n* = 6; NR)	12.4 mL/kg, IP (NR)	5 min
Cocilova 2019 [57]	United States	Turtle/T. scripta/adult (0.2–0.4 kg)/M and F	Brevetoxin-3	10.53 μg/kg (IT)	ILE (*n* = 6); control (*n* = 5; NR)	100 mg/kg, IV (NR)	30 min
Ghadiri 2017 [32]	Iran	Rat/Wistar/adult/M	Methamphetamine	45 mg/kg (IP)	ILE (*n* = 6); control (*n* = 6; saline)	Bolus: 18.6 mL/kg Intralipid 20%, IV (Fresenius Kabi)	5–7 min
Hori 2022 [58]	Japan	Rat/Sprague Dawley/adult/M	Sevoflurane	Induction: 3–5%; maintenance: 2% or 0.8%; 20 min	ILE (*n* = 6); control (*n* = 6; saline)	Bolus: 9 mL/kg, 1 min IV; CRI: 1.5 mL/kg/min, 4 min Intralipid 20%, IV (Fresenius Kabi)	<0.5 min
			Isoflurane	Induction: 2.5–4%; maintenance: 1.5%; 20 min			
Jesmok 1979 [59]	United States	Rat/Sprague Dawley/adult/M	Pentobarbital	30 mg/kg (IV)	ILE (*n* = 6/dose group); control (*n* = 15; saline)	Bolus: 0.16, 0.31, 1.25, or 2.5 g/kg, 30 s, Intralipid 10%, IV (Cutter Laboratories)	5 min
Kayipmaz 2014 [60]	Turkey	Rat/Sprague Dawley/adult/F	Methyl parathion	0.24 mg/kg (PO)	ILE (*n* = 7/dose group); control (*n* = 7)	Bolus: 3 mL/kg, NR, 20% lipid emulsion, IV (NR)	15 min
Li 2012 [38]	China	Rat/Sprague Dawley/adult/M and F	Dichlorvos	11 mg/kg (IP)	ILE (*n* = 12/dose group); control (*n* = 12; saline)	Bolus: 5 mL/kg, NR, 20% fat emulsion, IV (Shandong Huarui Pharmaceutical)	<0.5 min
Moshiri 2013 [42]	Iran	Rat/Sprague Dawley/adult/M	Diazinon	480 mg/kg (PO)	ILE 10% (*n* = 6); ILE 20% (*n* = 6); saline 16 mL/kg (*n* = 6); saline 10 mL/kg (*n* = 6)	Bolus: 10 mL/kg, 15 min, ILE 10%, or ILE 20%, IV (Fresenius Kabi)	30 min
Moshiri 2014 [43]	Iran	Rabbit/NZW/adult/M	Haloperidol (HAL)	2.6 mg/kg (IV)	Water + saline (*n* = 5); water + ILE 18 (*n* = 5); HAL + saline (*n* = 5); HAL + ILE 6, 12, or 18 (*n* = 15)	Bolus: 6, 12, or 18 mL/kg Intralipid 20%, IV (Fresenius Kabi)	30 min
Ozkan 2014 [61]	Turkey	Rat/Wistar/adult/F	Chlorpyriphos	10 mg/kg (PO)	ILE (*n* = 7); control (*n* = 7)	18.6 mL/kg, PO (NR)	<0.5 min
Vahabzadeh 2013 [48]	Iran	Rabbit/NZW/adult/M	Tramadol	50 mg/kg (IV)	ILE (*n* = 15, 5/group); control (*n* = 5; saline)	Bolus: 6, 12, or 18 mL/kg ILE 20%, IV (Fresenius Kabi AB)	30 min
Yousefsani 2019 [51]	Iran	Rat/Wistar/93–96 d/M	Clozapine	40 mg/kg (IV)	ILE (*n* = 6); control (*n* = 6; saline)	Bolus: 18.6 mL/kg ILE 20%, IV (Fresenius Kabi AB)	15 min

Abbreviations: F: female; IP: intraperitoneal; IV: intravenous, M: male; min: minute; NR: not reported; NZW: New Zealand White; PO: per os.

**Table 4:** Profile of confidence in the body of evidence on whether in animal models of toxicant poisoning treatment with ILE, compared with no ILE treatment, improves neurological outcomes. No upgrades occurred. Key: — if minimal or no concern was present; ↓ if serious concern to downgrade confidence by one point. A detailed rationale for judgments regarding risk of bias, indirectness, inconsistency, imprecision, and publication bias is provided in Supplemental Table S5.

		Downgrades					
Outcome	Initial Confidence Rating	Risk of Bias	Indirectness	Inconsistency	Imprecision	Publication Bias	Certainty Rating
Neuropathology	4	↓	↓	↓	↓	—	Very Low
8 experimental studies							
[28,32,43,52,53,54,60,61]							
6 rat, 2 rabbit							
Neurological signs	4	↓	—	↓	↓	—	Very Low
10 experimental studies							
[28,38,42,43,48,51,54,55,57,60]							
5 rat, 4 rabbit, 1 turtle							
2 RCTs							
[62,63]							
2 cat							
Behavior	4	↓	—	↓	↓	—	Very Low
3 studies							
[32,51,56]							
2 rat, 1 mouse							
Recovery from anesthesia	4	↓	—	—	↓	—	Low
2 studies							
[58,59]							
2 rat							

**Table 5:** Overview of randomized clinical trials.

Study	Country of Publication	Study Type	Species	Toxicant	Groups	ILE Dose, Route (Source)	Most Common Clinical Signs at Presentation	Main Findings
Lundgren 2024 [62]	Sweden	Prospective, randomized clinical study	Cat/various/0.25 to 20.5 y/M and F	Alpha-chloralose	ILE (*n* = 13); control (*n* = 12; Ringer’s acetate)	Bolus: 300 mg/kg, 2 min; CRI: 1500 mg/kg over 30 min (20% ILE, Fresenius Kabi)	Tremor (88%), cranial nerve deficits (80%), bradycardia (76%), visual impairment (44%), reduced PLR (44%), miosis (36%), mydriasis (8%)	All cats recovered within 72 h and survived until follow-up. Serum alpha-chloralose concentration after treatment and intoxication score ILE vs. control: NSD.
Peacock 2015 [63]	Australia	Randomized clinical trial	Cat Age, median years (1st quartile, 3rd quartile) 3.3 (1.3, 4.5), 4.0 (1.4, 5.1), 0.324	Permethrin	ILE (*n* = 20); control (*n* = 14; 0.9% saline); cats were excluded if they had received treatment for toxicosis prior to presentation	CRI: 15 mL/kg, 60 min (20% ILE, Baxter Healthcare)	Tremor, ataxia, lateral recumbency, seizures; recruited subjects were staged based on presence of these signs	Tremors treated with methocarbamol and seizures with diazepam. Dermal decontamination was performed on all cats. ILE-treated cats had lower clinical stages earlier than controls (*p* < 0.05). Signalment, body weight, or supportive treatment between the groups: NSD.

Abbreviations: CRI: constant rate infusion; ILE: intravenous lipid emulsion.

## Data Availability

No new data were created or analyzed in this study. Data sharing is not applicable to this article.

## Supplementary Materials

The following supporting information can be downloaded at https://www.mdpi.com/article/10.3390/vetsci13101059/s1, Supplemental File S1: Complete search strategies for PubMed, Web of Science, and CAB Abstracts. Figure S1: Forest plot analysis of the overall relative risk of death in ILE-treated animals following exposure to a tricyclic antidepressant; Figure S2: Forest plot analysis of the overall relative risk of death in ILE-treated animals following exposure to an organophosphate insecticide; Figure S3: Forest plot analysis of the overall relative risk of death in ILE-treated dogs, rats, and rabbits following toxicant exposure; Figure S4: Funnel plots for (A) the global mortality meta-analysis and (B) the exploratory mortality analysis, excluding swine studies. Table S1: Preferred Reporting Items for Systematic Reviews and Meta-Analyses (PRISMA 2020) checklist; Table S2: Mortality in experimental studies; Table S3: GRADE assessment of survival in animal toxicology studies evaluating intravenous lipid emulsions (ILE); Table S4. Neurological outcomes of interest in experimental studies; Table S5. GRADE assessment of neurological outcomes in animal toxicology studies evaluating ILE; Table S6. Excluded studies and reasons for exclusion.

## Abbreviations

The following abbreviations are used in this manuscript:

CPR	Cardiopulmonary resuscitation
CRI	Constant rate infusion
F	Female
ILE	Intravenous lipid emulsion
IP	Intraperitoneal
IV	Intravenous
GRADE	Grading of Recommendations, Assessment, Development, and Evaluation
LAST	Local anesthetic systemic toxicity
MAP	Mean arterial pressure
NR	Not reported
NSD	No significant difference
NZW	New Zealand white
PICO	Population, Intervention, Comparison, Outcome
PO	Per os
PRISMA	Preferred Reporting Items for Systematic Reviews and Meta-Analyses
RR	Relative risk
