# Supplementary material for: Lipid Emulsion as a Treatment for Non-Local Anesthetic Poisoning in Animals: A Systematic Review and Meta-Analysis of Survival and Neurological Outcomes
Source: Vet Sci. 2026 Oct 8;13(10):1059. doi: 10.3390/vetsci13101059 (PMC13709335; doi:10.3390/vetsci13101059)
Supplement: Supplementary file 1 [file vetsci-13-01059-s001.zip › vetsci-4500103-supplementary.pdf]

veterinary sciences

MDPI

Table S1: Preferred Reporting Items for Systematic Reviews and Meta-Analyses (PRISMA 2020) Checklist

Section and Topic	Item #	Checklist item	Location where item is reported
**TITLE**			
Title	1	Identify the report as a systematic review.	Manuscript title
**ABSTRACT**			
Abstract	2	See the PRISMA 2020 for Abstracts checklist.	Abstract
**INTRODUCTION**			
Rationale	3	Describe the rationale for the review in the context of existing knowledge.	Section 1 (Introduction)
Objectives	4	Provide an explicit statement of the objective(s) or question(s) the review addresses.	Section 1 (Introduction), review objectives and review question
**METHODS**			
Eligibility criteria	5	Specify the inclusion and exclusion criteria for the review and how studies were grouped for the syntheses.	Section 2.3 (Study Eligibility and the PICO Framework); Section 2.8 (Evidence Synthesis)
Information sources	6	Specify all databases, registers, websites, organisations, reference lists and other sources searched or consulted to identify studies. Specify the date when each source was last searched or consulted.	Section 2.2 (Search Strategy); Section 3.1 (Results of the Search), reference-list searching
Search strategy	7	Present the full search strategies for all databases, registers and websites, including any filters and limits used.	Section 2.2 (Search Strategy); Supplemental File S1 (complete database search strategies)

Section and Topic	Item #	Checklist item	Location where item is reported
Selection process	8	Specify the methods used to decide whether a study met the inclusion criteria of the review, including how many reviewers screened each record and each report retrieved, whether they worked independently, and if applicable, details of automation tools used in the process.	Section 2.4 (Study Screening); Section 2.3 (Study Eligibility and the PICO Framework), re-evaluation following peer review
Data collection process	9	Specify the methods used to collect data from reports, including how many reviewers collected data from each report, whether they worked independently, any processes for obtaining or confirming data from study investigators, and if applicable, details of automation tools used in the process.	Section 2.5 (Data Extraction); Section 2.6 (Quality Assessment), missing-data contact policy
Data items	10a	List and define all outcomes for which data were sought. Specify whether all results that were compatible with each outcome domain in each study were sought (e.g. for all measures, time points, analyses), and if not, the methods used to decide which results to collect.	Section 2.1 (Protocol and Registration); Section 2.3 (Study Eligibility and the PICO Framework); Sections 3.2–3.4 (survival, neurological outcomes, and randomized clinical trials)
	10b	List and define all other variables for which data were sought (e.g. participant and intervention characteristics, funding sources). Describe any assumptions made about any missing or unclear information.	Section 2.5 (Data Extraction); Section 2.6 (Quality Assessment), handling of unreported methods; Tables 1, 3, and 5
Study risk of bias assessment	11	Specify the methods used to assess risk of bias in the included studies, including details of the tool(s) used, how many reviewers assessed each study and whether they worked independently, and if applicable, details of automation tools used in the process.	Section 2.6 (Quality Assessment)
Effect measures	12	Specify for each outcome the effect measure(s) (e.g. risk ratio, mean difference) used in the synthesis or presentation of results.	Section 2.7 (Method of Analysis)

https://doi.org/10.3390/xxxxx

Section and Topic	Item #	Checklist item	Location where item is reported
Synthesis methods	13a	Describe the processes used to decide which studies were eligible for each synthesis (e.g. tabulating the study intervention characteristics and comparing against the planned groups for each synthesis (item #5)).	Section 2.3 (Study Eligibility and the PICO Framework); Section 2.7 (Method of Analysis); Section 2.8 (Evidence Synthesis)
	13b	Describe any methods required to prepare the data for presentation or synthesis, such as handling of missing summary statistics, or data conversions.	Section 2.5 (Data Extraction), graphical data extraction; Section 2.7 (Method of Analysis), zero-cell correction and pooling of shared-control arms
	13c	Describe any methods used to tabulate or visually display results of individual studies and syntheses.	Section 2.7 (Method of Analysis), forest plots; Section 2.8 (Evidence Synthesis), evidence profiles and summary-of-findings tables; Sections 3.2–3.4 and accompanying tables and figures
	13d	Describe any methods used to synthesize results and provide a rationale for the choice(s). If meta-analysis was performed, describe the model(s), method(s) to identify the presence and extent of statistical heterogeneity, and software package(s) used.	Section 2.7 (Method of Analysis); Section 2.8 (Evidence Synthesis); Section 4.2 (Neurological Outcomes in Poisoned Animals Following ILE Treatment),

https://doi.org/10.3390/xxxxx

Section and Topic	Item #	Checklist item	Location where item is reported
			rationale for qualitative synthesis
	13e	Describe any methods used to explore possible causes of heterogeneity among study results (e.g. subgroup analysis, meta-regression).	Section 2.7 (Method of Analysis), exploratory toxicant-specific subgroup analyses; Section 2.1 (Protocol and Registration), exploratory status; Section 4.1 (Survival Following ILE Treatment of Poisoned Animals), potential sources of heterogeneity
	13f	Describe any sensitivity analyses conducted to assess robustness of the synthesized results.	Section 2.7 (Method of Analysis), exploratory sensitivity analysis excluding swine; Section 2.1 (Protocol and Registration), exploratory status
Reporting bias assessment	14	Describe any methods used to assess risk of bias due to missing results in a synthesis (arising from reporting biases).	Section 2.7 (Method of Analysis), Egger's test for funnel plot asymmetry/small-study effects; Section 2.8 (Evidence Synthesis), publication bias in GRADE assessment

https://doi.org/10.3390/xxxxx

Section and Topic	Item #	Checklist item	Location where item is reported
Certainty assessment	15	Describe any methods used to assess certainty (or confidence) in the body of evidence for an outcome.	Section 2.8 (Evidence Synthesis)
**RESULTS**			
Study selection	16a	Describe the results of the search and selection process, from the number of records identified in the search to the number of studies included in the review, ideally using a flow diagram.	Section 3.1 (Results of the Search); Figure 1 (PRISMA diagram)
	16b	Cite studies that might appear to meet the inclusion criteria, but which were excluded, and explain why they were excluded.	Table S6 (Excluded studies and reasons for exclusion)
Study characteristics	17	Cite each included study and present its characteristics.	Sections 3.2–3.4; Tables 1, 3, and 5; Tables S2 and S4
Risk of bias in studies	18	Present assessments of risk of bias for each included study.	Sections 3.2–3.4, risk-of-bias assessments; Figures 3–5
Results of individual studies	19	For all outcomes, present, for each study: (a) summary statistics for each group (where appropriate) and (b) an effect estimate and its precision (e.g. confidence/credible interval), ideally using structured tables or plots.	Sections 3.2–3.4; Tables S2 and S4; Tables 1, 3, and 5; Figure 2 and Figures S1–S3
Results of syntheses	20a	For each synthesis, briefly summarise the characteristics and risk of bias among contributing studies.	Sections 3.2–3.4; Tables 1, 3, and 5; Figures 3–5
	20b	Present results of all statistical syntheses conducted. If meta-analysis was done, present for each the summary estimate and its precision (e.g. confidence/credible interval) and measures of statistical heterogeneity. If comparing groups, describe the direction of the effect.	Section 3.2 (Experimental Studies Evaluating Survival); Figure 2; Figures S1–S3
	20c	Present results of all investigations of possible causes of heterogeneity among study results.	Section 3.2 (Experimental Studies Evaluating Survival), toxicant-specific subgroup analyses and non-

Section and Topic	Item #	Checklist item	Location where item is reported
			swine analysis; Figures S1–S3
	20d	Present results of all sensitivity analyses conducted to assess the robustness of the synthesized results.	Section 3.2 (Experimental Studies Evaluating Survival), sensitivity analysis excluding swine; Figure S3
Reporting biases	21	Present assessments of risk of bias due to missing results (arising from reporting biases) for each synthesis assessed.	Section 3.2 (Experimental Studies Evaluating Survival), Egger's test results; Figure S4 (funnel plots); Tables S3 and S5 (publication-bias judgments)
Certainty of evidence	22	Present assessments of certainty (or confidence) in the body of evidence for each outcome assessed.	Sections 3.2–3.4; Tables 2 and 4; Tables S3 and S5
**DISCUSSION**			
Discussion	23a	Provide a general interpretation of the results in the context of other evidence.	Sections 4.1–4.2 (Discussion of survival and neurological outcomes)
	23b	Discuss any limitations of the evidence included in the review.	Sections 4.1–4.3 (Discussion and study limitations)
	23c	Discuss any limitations of the review processes used.	Section 4.3 (Study Limitations and Next Steps)
	23d	Discuss implications of the results for practice, policy, and future research.	Sections 4.1–4.3; Section 5 (Conclusions)
**OTHER INFORMATION**			

https://doi.org/10.3390/xxxxx

Section and Topic	Item #	Checklist item	Location where item is reported
Registration and protocol	24a	Provide registration information for the review, including register name and registration number, or state that the review was not registered.	Section 2.1 (Protocol and Registration), Open Science Framework registration
	24b	Indicate where the review protocol can be accessed, or state that a protocol was not prepared.	Section 2.1 (Protocol and Registration), protocol access link
	24c	Describe and explain any amendments to information provided at registration or in the protocol.	Section 2.1 (Protocol and Registration); Section 2.3 (Study Eligibility and the PICO Framework); Section 4.3 (Study Limitations and Next Steps)
Support	25	Describe sources of financial or non-financial support for the review, and the role of the funders or sponsors in the review.	Funding statement
Competing interests	26	Declare any competing interests of review authors.	Conflicts of Interest statement
Availability of data, code and other materials	27	Report which of the following are publicly available and where they can be found: template data collection forms; data extracted from included studies; data used for all analyses; analytic code; any other materials used in the review.	Data Availability Statement; Supplementary Materials statement (search strategies, extracted study data, and supporting analyses)

From: Page MJ, McKenzie JE, Bossuyt PM, Boutron I, Hoffmann TC, Mulrow CD, et al. The PRISMA 2020 statement: an updated guideline for reporting systematic reviews. BMJ 2021;372:n71. doi: 10.1136/bmj.n71.

Supplemental File S1. Complete search strategies for PubMed, Web of Science, and CAB Abstracts

PubMed:

("pharmaceutical preparations"[mesh] OR drug[tw] OR drugs[tw] OR pharmaceutical[tw] OR pharmaceuticals[tw] OR "toxins, biological"[Mesh] OR "toxicology"[Mesh] OR "toxin"[tw] OR "toxins"[tw] OR "toxicity"[tw] OR "toxicology"[tw] OR "toxicologies"[tw] OR "intoxication"[tw] OR "intoxications"[tw] OR "intoxicated"[tw] OR "toxicosis"[tw] OR "toxidrome"[tw] OR "poisoning"[Mesh] OR "poisoning"[tw] OR "poisonings"[tw] OR "poison"[tw] OR "poisons"[tw] OR "exposure"[tw] OR "exposed"[tw] OR "exposures"[tw] OR "exposing"[tw] OR "drug overdose"[Mesh] OR "overdose"[tw] OR "overdoses"[tw] OR "overdosed"[tw] OR "reversal"[tw] OR "amitriptyline"[Mesh] OR "amitriptyline"[tw] OR "elavil"[tw] OR "amlodipine"[Mesh] OR "amlodipine"[tw] OR "norvasc"[tw] OR "katerzia"[tw] OR "baclofen"[Mesh] OR "baclofen"[tw] OR "kemstro"[tw] OR "lioresal"[tw] OR "gablofen"[tw] OR "brevetoxin"[Supplementary Concept] OR "brevetoxin"[tw] OR "bromethalin"[Supplementary Concept] OR "bromethalin"[tw] OR "chocolate"[Mesh] OR "chocolate"[tw] OR "chocolates"[tw] OR "chloralose"[Mesh] OR "chloralose"[tw] OR "clozapine"[Mesh] OR "clozapine"[tw] OR "clozaril"[tw] OR "fazaclo"[tw] OR "versacloz"[tw] OR "cocaine"[Mesh] OR "cocaine"[tw] OR "cyclobenzaprine"[Supplementary Concept] OR "cyclobenzaprine"[tw] OR "flexeril"[tw] OR "amrix"[tw] OR "fexmid"[tw] OR "diazepam"[Mesh] OR "diazepam"[tw] OR "valtoco"[tw] OR "diastat"[tw] OR "valium"[tw] OR "diazinon"[Mesh] OR "diazinon"[tw] OR "disulfoton"[Mesh] OR "disulfoton"[tw] OR "dronabinol"[Mesh] OR "dronabinol"[tw] OR "marinol"[tw] OR "etomidate"[Mesh] OR "etomidate"[tw] OR "amidate"[tw] OR "fipronil"[Supplementary Concept] OR "fipronil"[tw] OR "frontline"[tw] OR "fluorouracil"[Mesh] OR "fluorouracil"[tw] OR "carac"[tw] OR "efudex"[tw] OR "tolak"[tw] OR "adrucil"[tw] OR "fluoroplex"[tw] OR "imidacloprid"[Supplementary Concept] OR "imidacloprid"[tw] OR "advantage multi"[tw] OR "advantix"[tw] OR "provecta"[tw] OR "isoflurane"[Mesh] OR "isoflurane"[tw] OR "forane"[tw] OR "terrell"[tw] OR "isoniazid"[Mesh] OR "isonazid"[tw] OR "ivermectin"[Mesh] OR "ivermectin"[tw] OR "stromectol"[tw] OR "sklice"[tw] OR "soolantra"[tw] OR "lamotrigine"[Mesh] OR "lamotrigine"[tw] OR "lamictal"[tw] OR "lisdexamfetamine dimesylate"[Mesh] OR "lisdexamfetamine dimesylate"[tw] OR "metaldehyde"[Supplementary Concept] OR "metaldehyde"[tw] OR "methamphetamine"[Mesh] OR "methamphetamine"[tw] OR "desoxyn"[tw] OR "methomyl"[Mesh] OR "methomyl"[tw] OR "morphine"[Mesh] OR "morphine"[tw] OR "duramorph"[tw] OR "avinza"[tw] OR "roxanol"[tw] OR "ms-contin"[tw] OR "statex"[tw] OR "kadian"[tw] OR "morphabond"[tw] OR "arymo"[tw] OR "infumorph"[tw] OR "depodur"[tw] OR "moxidectin"[Supplementary Concept] OR "moxidectin"[tw] OR "pentobarbital"[Mesh] OR "pentobarbital"[tw] OR "nembutal"[tw] OR "permethrin"[Mesh] OR "permethrin"[tw] OR "elimite"[tw] OR "acticin"[tw] OR "phenobarbital"[Mesh] OR "phenobarbital"[tw] OR "praziquantel"[Mesh] OR "praziquantel"[tw] OR "biltricide"[tw] OR "sevoflurane"[Mesh] OR "sevoflurane"[tw] OR "ultane"[tw] OR "sojourn"[tw] OR "thiopental"[Mesh] OR "thiopental"[tw] OR "pentothal"[tw] OR "tramadol"[Mesh] OR "tramadol"[tw] OR "ultram"[tw] OR "ultracet"[tw] OR "conzip"[tw] OR "ryzolt"[tw] OR "qdolo"[tw]) AND ("fat emulsions, intravenous"[Mesh] OR "fat emulsions, intravenous"[Pharmacological Action] OR "lipid emulsions"[tw] OR "lipid emulsion"[tw] OR "lipid injectable"[tw] OR "fat emulsions"[tw] OR "fat emulsion"[tw] OR "lipid rescue"[tw] OR "lipid resuscitation"[tw] OR "intravenous lipid"[tw] OR intralipid[tw] OR nutrilipid[tw] OR smoflipid[tw] OR clinolipid[tw] OR omegaven[tw] OR liposyn[tw]) AND (("Mammals"[mesh] OR mammal[tw] OR mammals[tw] OR mammalia[tw]) NOT (Humans[mesh] OR human[tw] OR humans[tw] OR "homo sapiens"[tw])) OR (animals[tw] OR animal[tw] OR mice[tw] OR mus[tw] OR mouse[tw] OR murine[tw] OR woodmouse[tw] OR rats[tw] OR rat[tw] OR murinae[tw] OR muridae[tw] OR cottonrat[tw] OR cottonrats[tw] OR

hamster[tw] OR hamsters[tw] OR cricetinae[tw] OR porcupine[tw] OR porcupines[tw] OR rodentia[tw] OR rodent[tw] OR rodents[tw] OR pigs[tw] OR pig[tw] OR swine[tw] OR swines[tw] OR piglets[tw] OR piglet[tw] OR boar[tw] OR boars[tw] OR "sus scrofa"[tw] OR ferrets[tw] OR ferret[tw] OR polecat[tw] OR polecats[tw] OR "mustela putorius"[tw] OR "guinea pigs"[tw] OR "guinea pig"[tw] OR cavia[tw] OR callithrix[tw] OR marmoset[tw] OR marmosets[tw] OR cebuella[tw] OR hapale[tw] OR octodon[tw] OR chinchilla[tw] OR chinchillas[tw] OR gerbillinae[tw] OR gerbil[tw] OR gerbils[tw] OR jird[tw] OR jirds[tw] OR merione[tw] OR meriones[tw] OR rabbits[tw] OR rabbit[tw] OR hares[tw] OR hare[tw] OR cats[tw] OR cat[tw] OR carus[tw] OR felis[tw] OR lion[tw] OR lions[tw] OR tiger[tw] OR tigers[tw] OR panther[tw] OR panthers[tw] OR leopard[tw] OR leopards[tw] OR felidae[tw] OR dogs[tw] OR dog[tw] OR canine[tw] OR canines[tw] OR canis[tw] OR wolf[tw] OR wolves[tw] OR fox[tw] OR foxes[tw] OR coyote[tw] OR coyotes[tw] OR canidae[tw] OR hyena[tw] OR hyenas[tw] OR sheep[tw] OR sheeps[tw] OR mouflon[tw] OR mouflons[tw] OR ovis[tw] OR goats[tw] OR goat[tw] OR capra[tw] OR capras[tw] OR rupicapra[tw] OR chamois[tw] OR haplorhini[tw] OR monkey[tw] OR monkeys[tw] OR anthropoidea[tw] OR anthropoids[tw] OR saguinus[tw] OR tamarin[tw] OR tamarins[tw] OR leontopithecus[tw] OR hominidae[tw] OR ape[tw] OR apes[tw] OR pan[tw] OR paniscus[tw] OR bonobos[tw] OR troglodytes[tw] OR "pan troglodytes"[tw] OR baboon[tw] OR baboons[tw] OR gibbon[tw] OR gibbons[tw] OR siamang[tw] OR siamangs[tw] OR nomascus[tw] OR symphalangus[tw] OR chimpanzee[tw] OR chimpanzees[tw] OR capuchin[tw] OR capuchins[tw] OR prosimians[tw] OR "bush baby"[tw] OR prosimian[tw] OR bush babies[tw] OR galagos[tw] OR galago[tw] OR pongidae[tw] OR gorilla[tw] OR gorillas[tw] OR pongo[tw] OR pygmaeus[tw] OR "pongo pygmaeus"[tw] OR orangutans[tw] OR pygmaeus[tw] OR lemur[tw] OR lemurs[tw] OR lemuridae[tw] OR horse[tw] OR horses[tw] OR pongo[tw] OR equus[tw] OR cow[tw] OR calf[tw] OR bull[tw] OR sciuridae[tw] OR squirrel[tw] OR squirrels[tw] OR chipmunk[tw] OR chipmunks[tw] OR hedgehog[tw] OR hedgehogs[tw] OR suslik[tw] OR susliks[tw] OR mole[tw] OR moles[tw] OR talpidae[tw] OR vole[tw] OR voles[tw] OR lemming[tw] OR lemmings[tw] OR muskrat[tw] OR muskrats[tw] OR lemmus[tw] OR otter[tw] OR otters[tw] OR marten[tw] OR martens[tw] OR martes[tw] OR weasel[tw] OR badger[tw] OR badgers[tw] OR ermine[tw] OR mink[tw] OR minks[tw] OR sable[tw] OR sables[tw] OR gulo[tw] OR gulos[tw] OR wolverine[tw] OR wolverines[tw] OR mustela[tw] OR armadillo[tw] OR armadillos[tw] OR sloth[tw] OR sloths[tw] OR anteater[tw] OR anteaters[tw] OR llama[tw] OR llamas[tw] OR alpaca[tw] OR alpacas[tw] OR camelid[tw] OR camelids[tw] OR guanaco[tw] OR guanacos[tw] OR chiroptera[tw] OR chiropteras[tw] OR bat[tw] OR bats[tw] OR fox[tw] OR foxes[tw] OR donkey[tw] OR donkeys[tw] OR mule[tw] OR mules[tw] OR zebra[tw] OR zebras[tw] OR shrew[tw] OR shrews[tw] OR bison[tw] OR bisons[tw] OR buffalo[tw] OR buffaloes[tw] OR deer[tw] OR deers[tw] OR giraffe[tw] OR giraffes[tw] OR bear[tw] OR bears[tw] OR panda[tw] OR pandas[tw] OR "wild hog"[tw] OR "wild boar"[tw] OR fitchew[tw] OR fitch[tw] OR beaver[tw] OR beavers[tw] OR jerboa[tw] OR jerboas[tw] OR capybara[tw] OR capybaras[tw] OR elephant[tw] OR elephants[tw] OR elephantidae[tw] OR wombat[tw] OR wombats[tw] OR koala[tw] OR koalas[tw] OR opossum[tw] OR opossums[tw] OR possum[tw] OR possums[tw] OR didelphis[tw] OR didelphidae[tw] OR kangaroo[tw] OR kangaroos[tw] OR wallaby[tw] OR wallabies[tw] OR macropodidae[tw] OR seal[tw] OR seals[tw] OR walrus[tw] OR walruses[tw] OR pinniped[tw] OR pinnipeds[tw] OR desmatophocidae[tw])

Web of Science and CAB Abstracts:

("pharmaceutical preparations" OR drug OR drugs OR pharmaceutical OR pharmaceuticals OR toxicology OR toxin OR toxins OR toxicity OR toxicology OR toxicologies OR intoxication OR intoxications OR intoxicated OR toxicosis OR toxidrome OR poisoning OR poisoning OR poisonings OR poisonOR poisons OR exposure

https://doi.org/10.3390/xxxxx

OR exposed OR exposures OR exposing OR overdose OR overdoses OR overdosed OR reversal OR amitriptyline OR elavil OR amlodipine OR norvasc OR katerzia OR baclofen OR kemstro OR lioresal OR gablofen OR brevetoxin OR bromethalin OR chocolate OR chocolates OR chloralose OR clozapine OR clozaril OR fazaclo OR versacloz OR cocaine OR cyclobenzaprine OR flexeril OR amrix OR fexmid OR diazepam OR valtoco OR diastat OR valium OR diazinon OR disulfoton OR dronabinol OR marinol OR etomidate OR amidate OR fipronil OR frontline OR fluorouracil OR carac OR efudex OR tolak OR adrucil OR fluoroplex OR imidacloprid OR "advantage multi" OR advantix OR provecta OR isoflurane OR forane OR terrell OR isoniazid OR ivermectin OR stromectol OR sklice OR soolantra OR lamotrigine OR lamictal OR "lisdexamfetamine dimesylate" OR metaldehyde OR methamphetamine OR desoxyn OR methomyl OR morphine OR duramorph OR avinza OR roxanol OR ms-contin OR statex OR kadian OR morphabond OR arymo OR infumorph OR depodur OR moxidectin OR pentobarbital OR nembutal OR permethrin OR elimite OR acticin OR phenobarbital OR praziquantel OR biltricide OR sevoflurane OR ultane OR sojourn OR thiopental OR pentothal OR tramadol OR ultram OR ultracet OR conzip OR ryzolt OR qdolo) AND ("lipid emulsions" OR "lipid emulsion" OR "lipid injectable" OR "fat emulsions" OR "fat emulsion" OR "lipid rescue" OR "lipid resuscitation" OR "intravenous lipid" OR intralipid OR nutrilipid OR smoflipid OR clinolipid OR omegaven OR liposyn) AND ((mammal OR mammals OR mammalia) NOT (human OR humans OR "homo sapiens")) OR (animals OR animal OR mice OR mus OR mouse OR murine OR woodmouse OR rats OR rat OR murinae OR muridae OR cottonrat OR cottonrats OR hamster OR hamsters OR cricetinae OR porcupine OR porcupines OR rodentia OR rodent OR rodents OR pigs OR pig OR swine OR swines OR piglets OR piglet OR boar OR boars OR "sus scrofa" OR ferrets OR ferret OR polecat OR polecats OR "mustela putorius" OR "guinea pigs" OR "guinea pig" OR cavia OR callithrix OR marmoset OR marmosets OR cebuella OR hapale OR octodon OR chinchilla OR chinchillas OR gerbillinae OR gerbil OR gerbils OR jird OR jirds OR merione OR meriones OR rabbits OR rabbit OR hares OR hare OR cats OR cat OR carus OR felis OR lion OR lions OR tiger OR tigers OR panther OR panthers OR leopard OR leopards OR felidae OR dogs OR dog OR canine OR canines OR canis OR wolf OR wolves OR fox OR foxes OR coyote OR coyotes OR canidae OR hyena OR hyenas OR sheep OR sheeps OR mouflon OR mouflons OR ovis OR goats OR goat OR capra OR capras OR rupicapra OR chamois OR haplorhini OR monkey OR monkeys OR anthropoidea OR anthropoids OR saguinus OR tamarin OR tamarins OR leontopithecus OR hominidae OR ape OR apes OR pan OR paniscus OR bonobos OR troglodytes OR "pan troglodytes" OR baboon OR baboons OR gibbon OR gibbons OR siamang OR siamangs OR nomascus OR symphalangus OR chimpanzee OR chimpanzees OR capuchin OR capuchins OR prosimians OR "bush baby" OR prosimian OR "bush babies" OR galagos OR galago OR pongidae OR gorilla OR gorillas OR pongo OR pygmaeus OR "pongo pygmaeus" OR orangutans OR pygmaeus OR lemur OR lemurs OR lemuridae OR horse OR horses OR pongo OR equus OR cow OR calf OR bull OR sciuridae OR squirrel OR squirrels OR chipmunk OR chipmunks OR hedgehog OR hedgehogs OR suslik OR susliks OR mole OR moles OR talpidae OR vole OR voles OR lemming OR lemmings OR muskrat OR muskrats OR lemmus OR otter OR otters OR marten OR martens OR martes OR weasel OR badger OR badgers OR ermine OR mink OR minks OR sable OR sables OR gulo OR gulos OR wolverine OR wolverines OR mustela OR armadillo OR armadillos OR sloth OR sloths OR anteater OR anteaters OR llama OR llamas OR alpaca OR alpacas OR camelid OR camelids OR guanaco OR guanacos OR chiroptera OR chiropteras OR bat OR bats OR fox OR foxes OR donkey OR donkeys OR mule OR mules OR zebra OR zebras OR shrew OR shrews OR bison OR bisons OR buffalo OR buffaloes OR deer OR deers OR giraffe OR giraffes OR bear OR bears OR panda OR pandas OR "wild hog" OR "wild boar" OR fitchew OR fitch OR beaver OR beavers OR jerboa OR jerboas OR capybara OR capybaras OR elephant OR elephants OR elephantidae OR wombat OR wombats OR

https://doi.org/10.3390/xxxxx

koala OR koalas OR opossum OR opossums OR possum OR possums OR didelphis OR didelphidae OR kangaroo OR kangaroos OR wallaby OR wallabies OR macropodidae OR seal OR seals OR walrus OR walruses OR pin

https://doi.org/10.3390/xxxxx

Table S2. Mortality in experimental studies

Study	Species	Toxicant	Duration of monitoring after ILE administration	Findings	Additional Comments
Bania 2007 [26]	Dog	Verapamil	2 h	Survived: Control: 1/7; ILE: 7/7 (p = 0.32)	Dogs anesthetized with pentobarbital and isoflurane prior to verapamil administration. Dogs also given IV atropine, calcium chloride, and saline prior to ILE.
Cave 2013 [27]	Rabbit	Clomipramine	30 min	Survived: Control: 2/5; ILE: 5/5; (p = 0.05)	Rabbits were anesthetized with ketamine and xylazine and given vecuronium bromide prior to clomipramine administration.
Celikel 2014 [28]	Rat	Malathion	8 h	Survived: Control: 0/6; ILE: 0/6; (p = 1.0)	No deaths seen in rats given atropine and pralidoxime (+/- ILE)
Dunn 2012 [29]	Rat	Parathion	120 min	Survived: Control: 0/6; ILE5 min: 0/6; (p = 1.0)<br>Survived: Control: 0/6; ILE20 min: 4/6; (p = 0.02)	Rats were anesthetized with isoflurane prior to parathion administration. Respiratory arrest was a surrogate for survival. Lethal dose of parathion was given.
Fulton 2016a [30]	Pig	Venlafaxine	40 min	Survived: Control: 2/7; ILE: 2/7; (p = 1.0)	Pigs were anesthetized with telezol, glycopyrrolate, and isoflurane prior to venlafaxine administration. When pig developed a nonperfusing rhythm, CPR was initiated, isoflurane terminated, and midazolam and buprenex were given IV. Additional treatment groups not included here. Controls given CPR.
Fulton 2016b [31]	Pig	Bupropion	40 min	Survived: Control: 0/7; ILE: 0/7; (p = 1.0)	Pigs were premedicated with acepromazine, ketamine, and atropine and anesthetized with isoflurane prior to bupropion administration. When pig developed a nonperfusing rhythm, CPR was initiated, isoflurane terminated, and buprenex were

					given IV. Additional treatment groups not included here. Controls given CPR.
Ghadiri 2017 [32]	Rat	Methamphetamine	24 h	Survived: Control: 0/6; ILE: 3/6; (p = 0.06)	Controls given CPR.
Goor 2003 [33]	Rat	Clomipramine	24 h	Survived: Control: 0/9; ILE: 12/15; (p = 0.002)	Clomipramine and ILE co-administered
Harvey and Cave 2007 [34]	Rabbit	Clomipramine	15 min	Survived: Control: 0/4; ILE: 4/4; (p = 0.008)	Rabbits were anesthetized with ketamine and xylazine and given vecuronium bromide prior to propranolol administration.
Harvey 2014 [35]	Rabbit	Clomipramine	90 min	Survived: Control: 0/5; ILE: 2/5; (p = 0.13)	Rabbits were anesthetized with ketamine and given vecuronium bromide prior to clomipramine administration. Data for bicarbonate are not included here.
Heinonen 2013 [36]	Pig	Amitriptyline	75 min	Survived: Control: 10/10; ILE: 10/10; (p = 1.0)	Plasma total amitriptyline concentrations at 20 to 75 min: ILE (+) > ILE (-); p < 0.03. Mean (± SD) brain amitriptyline concentration ILE (+) 11.5 ± 2.8) mg/kg; ILE (-): 15.3 ± 4.5 mg/kg, p = 0.038.
Kara 2020 [37]	Rabbit	Clomipramine	120 min	Survived: Control: 6/6; ILE: 6/6; (p = 1.0)	Rabbits were anesthetized with ketamine and xylazine prior to clomipramine administration. Median plasma clomipramine concentration at 120 min: ILE (+): 28.55 µg/L; ILE (-): 14.5 µg/L; NSD. Data for bicarbonate and glucagon treatment groups are not included here.
Li 2012 [38]	Rat	Dichlorvos	24 h	No additional treatment: Survived: Control: 1/12; ILE: 2/12; (p = 0.54)<br><br>Treated with atropine (10 mg/kg, IM) and pralidoxime chloride (40 mg/kg, IM): Survived: Control: 6/12; ILE: 11/12; (p = 0.028)	None

https://doi.org/10.3390/xxxxx

Litonius 2012 [39]	Pig	Amitriptyline	60 min	Survived: Control: 18/20; ILE: 15/20; (p = 0.22)	Pigs were anesthetized with ketamine and isoflurane prior to amitriptyline administration.
Macala 2014 [40]	Rat	Clonidine	30 min	Survived: Control: 0/8; ILE: 7/8; (p = 0.006)	Rats were anesthetized with isoflurane prior to clonidine administration. Rats were also treated with propranolol (15 to 20 mg/kg, IV).
Macala 2018 [41]	Rat	Propranolol	60 min	Survived: Control: 0/4; ILE: 18/21; (p = 0.006)	Rats were anesthetized with isoflurane prior to propranolol administration. Survival rate rats treated with 20% ILE is pooled across three dose groups (1, 2, and 3 ml/kg).
Moshiri 2013 [42]	Rat	Diazinon	48 h	Survived: Control: 6/12; ILE: 11/12; (p = 0.028)	Data from other groups not included here
Moshiri 2014 [43]	Rabbit	Haloperidol	24 h	Survived: Control: 5/5; ILE 6: 5/5; (p = 1.0); ILE 12: 5/5; (p = 1.0); ILE 18: 3/5; (p = 0.13)	ILE 20% solution with 6, 12, and 18 mL/kg doses (ILE 6, ILE12, and ILE18 groups).
Nafea and Hassan 2019 [44]	Rat	Amlodipine	2 h	Survived: Control: 3/16; ILE: 14/16; (p = 0.0014)	Reanalyzed data to compare ILE versus control given amlodipine and saline. Data for no treatment control and methylene blue not included here.
O'Sullivan 2014 [45]	Pig	Desipramine	40 min	Survived: Control: 0/7; ILE: 2/7 (p = 0.14)	Pigs were anesthetized with telezol, glycopyrrolate, and isoflurane prior to desipramine administration. When pig developed a nonperfusing rhythm, CPR was initiated, isoflurane terminated, and midazolam and buprenex were given IV. Additional treatment groups not included here.
Perichon 2013 [46]	Rat	Amitriptyline	120 min	Survived: Control: 7/10; ILE: 1/10 (p = 0.005)	Rats were anesthetized with isoflurane and pentobarbital prior to amitriptyline administration. Data for negative control not included here. Controls given bicarbonate.
Şahin 2016 [47]	Rat	Verapamil	60	Survived: Control: 2/6; ILE: 4/6 (p = 0.268)	Rats were anesthetized with ketamine prior to verapamil administration. Data for medialipid 20% not included here. Manuscript appears to have reversed incidence data – extracted % survival data here.

Vahabzadeh 2013 [48]	Rabbit	Tramadol	24 h	Survived: Control: 3/5; ILE 6: 5/5; (p = 0.13); ILE 12: 5/5; (p = 0.13); ILE 18: 4/5; (p = 0.51)	Data for ILE only (no tramadol) group was not included. Note post hoc analysis of data was performed.
Varney 2014 [49]	Pig	Amitriptyline	60 min	Survived: Control: 4/12; ILE = 1/12 (p = 0.13)	Pigs were anesthetized with isoflurane prior to amitriptyline administration. Controls given bicarbonate.
Varney 2016 [50]	Pig	Diphenhydramine	60 min	Survived: Control: 2/12; ILE = 1/12 (p = 0.14)	Pigs were anesthetized with ketamine and isoflurane prior to diphenhydramine administration.
Yousefsani 2019 [51]	Rat	Clozapine	24 h	Survived: Control: 2/6; ILE = 0/6 (p = 0.14)	None

Abbreviations: CPR: cardiopulmonary resuscitation, ILE: intravenous lipid emulsion, IV: intravenous

https://doi.org/10.3390/xxxxx

veterinary sciences

Study	Intervention	Controls	Rel. Risk	95% CI	Weight (%)
Cave 2013	0/5	3/5	0.143	0.00924 to 2.209	3.93
Goor 2003	3/15	9/9	0.230	0.0902 to 0.588	13.37
Harvey and Cave 2007	0/4	4/4	0.111	0.00788 to 1.566	4.15
Harvey 2014	3/5	5/5	0.636	0.310 to 1.305	15.39
Heinonen 2013	0/10	5/10	0.100	0.00625 to 1.599	3.85
Kara 2020	0/6	0/6	1.000	0.0234 to 42.654	2.31
Litonius 2012	5/20	2/20	2.500	0.548 to 11.410	8.81
O'Sullivan 2014	5/7	7/7	0.733	0.445 to 1.209	17.30
Perichon 2013	9/10	3/10	3.000	1.138 to 7.906	13.08
Varney 2014	11/12	8/12	1.375	0.890 to 2.124	17.81
Total (random effects)	36/94	46/88	0.745	0.406 to 1.367	100

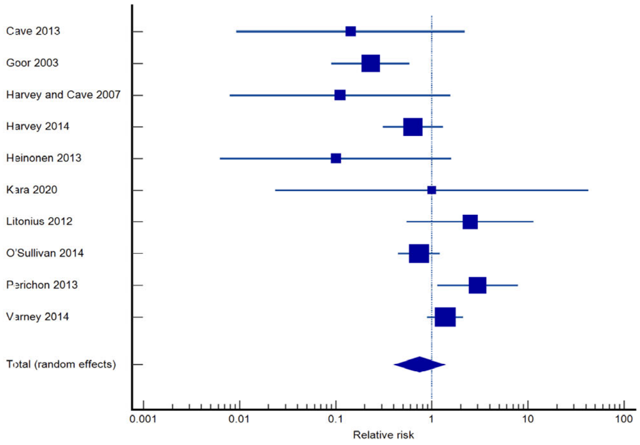

Heterogeneity: I^2 = 69.34%; Q 129.35; 95% CI 40.99 to 84.07; df = 9; P = 0.006
Test for overall effect: Z = −1.837; P = 0.066
Test for publication bias (Egger's test): Intercept = −1.049; 95% CI −3.25 to 1.16; P = 0.304

Figure S1. Forest plot analysis of the overall relative risk of death in ILE-treated animals following exposure to a tricyclic antidepressant. [27, 33, 34, 35, 36, 37, 39, 45, 46, 49]

Study	Intervention	Controls	Rel. Risk	95% CI	Weight (%)
Celikel 2014	6/6	6/6			
Dunn 2012	8/12	6/6	0.704	0.451 to 1.100	42.02
Li 2012	11/12	11/12	1.000	0.786 to 1.273	47.65
Moshiri 2013	1/12	6/12	0.167	0.0235 to 1.183	10.33
Total (random effects)	26/42	29/36	0.717	0.354 to 1.454	100

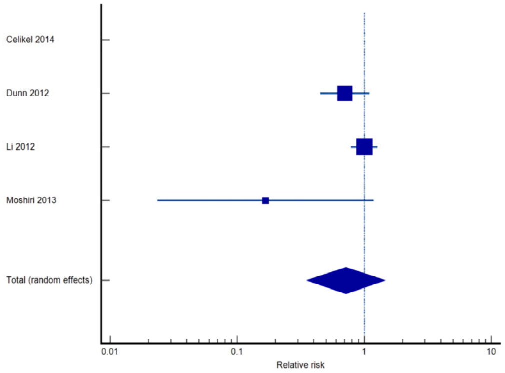

Heterogeneity: I^2 = 80.25%; Q 10.13; 95% CI 37.72 to 93.74; df = 2; P = 0.0063
Test for overall effect: z = −0.922; P = 0.356
Test for publication bias (Egger's test): Intercept = −2.26; 95% CI −8.94 to 4.43; P = 0.146

Figure S2. Forest plot analysis of the overall relative risk of death in ILE-treated animals following exposure to an organophosphate insecticide. [28, 29, 38, 42]

Study	Intervention	Controls	Rel. Risk	95% CI	Weight (%
Bania 2007	0/7	6/7	0.0769	0.00515 to 1.150	2.21
Cave 2013	0/5	3/5	0.143	2.16	2.68
Celikel 2014	6/6	6/6			
Dunn 2012	8/12	6/6	0.704	0.451 to 1.100	8.85
Ghadiri 2017	3/6	6/6	0.538	0.250 to 1.162	7.59
Goor 2003	3/15	9/9	0.230	0.0902 to 0.588	6.87
Harvey and Cave 2007	0/4	4/4	0.111	0.00788 to 1.566	2.28
Harvey 2014	3/5	5/5	0.636	0.310 to 1.305	7.80
Kara 2020	0/6	0/6	1.000	0.0234 to 42.654	1.29
Li 2012	11/12	11/12	1.000	0.786 to 1.273	9.411
Macala 2014	1/8	8/8	0.176	0.0406 to 0.767	4.83
Macala 2018	3/21	4/4	0.177	0.0648 to 0.486	6.59
Moshiri 2013	1/12	6/12	0.167	0.0235 to 1.183	3.48
Moshiri 2014	1/15	0/5	1.125	0.0527 to 23.995	1.81
Nafea and Hassan 2019	2/16	13/16	0.154	0.0412 to 0.575	5.36
Perichon 2013	9/10	3/10	3.000	1.138 to 7.906	6.74
Şahin 2016	2/6	4/6	0.500	0.141 to 1.772	5.56
Vahabzadeh 2013	2/15	2/5	0.333	0.0622 to 1.786	4.20
Yousefsani 2019	6/6	4/6	1.444	0.801 to 2.604	8.32
Total (random effects)	66/207	102/158	0.516	0.326 to 0.819	100

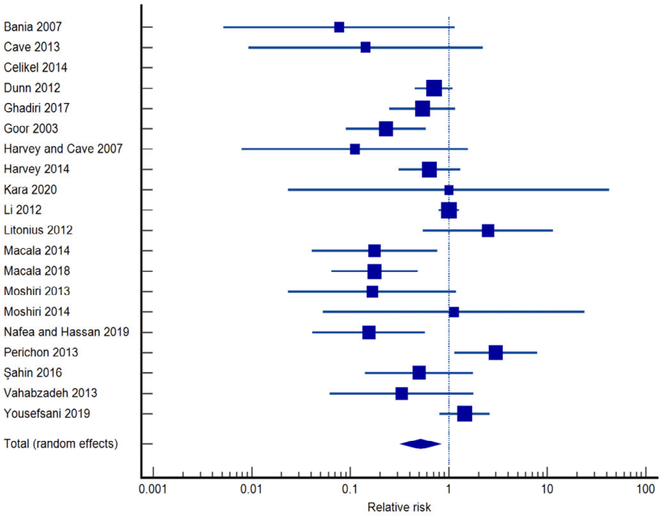

Heterogeneity: $I^2 = 75.85\%$; Q 74.55; 95% CI 62.41 to 84.49; df = 18; P < 0.0001
Test for overall effect: Z = −2.812; P = 0.005
Test for publication bias (Egger's test): Intercept = −1.3461; 95% CI −2.476 to −0.216; P = 0.0223

Figure S3. Forest plot analysis of the overall relative risk of death in ILE-treated dogs, rats, and rabbits following toxicant exposure. [26, 27, 28, 29, 32, 33, 34, 35, 37, 38, 40, 41, 42, 43, 44, 46, 47, 48, 51]

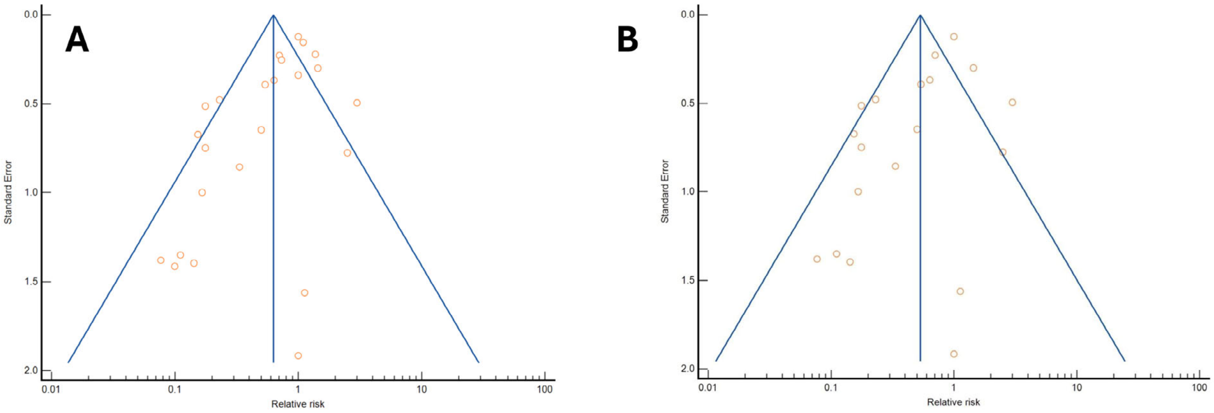

Figure S4: Funnel plots for (A) the global mortality meta-analysis and (B) the exploratory mortality analysis excluding swine studies.

Table S3. GRADE assessment of survival in animal toxicology studies evaluating intravenous lipid emulsions (ILE)

Domain	Assessment	Rationale
Risk of bias	Serious	Most studies demonstrated important methodological limitations. Randomization and allocation concealment were rarely described, and blinding of investigators or outcome assessors was often absent or unclear. Many experiments used highly controlled laboratory conditions with anesthetized animals, concurrent supportive therapies (e.g., atropine, bicarbonate, pralidoxime, CPR), or surrogate mortality endpoints, which may confound the independent effect of ILE. Sample sizes were uniformly small, often involving only 4–20 animals per group, increasing susceptibility to chance findings and exaggerated treatment effects.
Inconsistency	Serious	Results across studies were highly inconsistent. Some studies demonstrated substantial survival benefit with ILE (e.g., clomipramine, amlodipine, diazinon, dichlorvos with atropine/pralidoxime, delayed parathion treatment), whereas others showed no benefit or even potential harm (e.g., amitriptyline, diphenhydramine, clozapine). Effect direction and magnitude varied considerably across toxicants, species, timing of ILE administration, and co-interventions. Statistical heterogeneity was substantial. This inconsistency limits confidence in a generalized mortality benefit of ILE.
Indirectness	Possible	Many animals were anesthetized and mechanically supported, conditions that differ substantially from animal overdose scenarios. Toxicant doses were often supralethal or administered intravenously rather than through clinically relevant exposure routes. Monitoring periods were also short in several studies (15–120 minutes), limiting assessment of delayed mortality or recurrent toxicity. Consequently, clinical applicability to animal poisoning management is uncertain.
Imprecision	Serious	Confidence intervals around effect estimates were wide because of small sample sizes and low event counts. Numerous studies were underpowered to detect clinically meaningful mortality differences, and several statistically nonsignificant findings may reflect type II error rather than absence of effect. Conversely, some dramatic positive findings derived from very small experiments (e.g., 0/4 vs 4/4 survival), which may overestimate treatment effects.
Publication bias	Serious	The evidence base consists primarily of small experimental studies with positive or exploratory findings. Negative or neutral animal studies may be underreported. The result of a global (all mortality studies) meta-analysis of the relative risk (RR) of mortality following toxicant exposure followed by ILE administration demonstrated significant funnel-plot asymmetry, supporting concern for publication bias (Egger's test; P = 0.0048).

https://doi.org/10.3390/xxxxx

Table S4. Neurologic outcomes of interest in experimental studies

Study	Species	Toxicant	Neurologic outcomes of interest	Duration of monitoring after ILE administration	Neurologic outcome findings	Additional Comments
Basarslan 2014 [52]	Rat/NR/NR/NR	Malathion	Caspase-3 immunohistochemistry	0, 6, or 12 h	The number of caspase-3-positive brain cells in rats given malathion and ILE were increased versus untreated controls ($p = 0.001$).	Brain total antioxidant capacity and total oxidant status levels were also determined but not reported here. Quantitative caspase data was not provided, unknown whether ILE had a treatment effect.
Basarslan 2023 [53]	Rat/Wistar/adult/F	2-Glyphosate isopropylamine	Caspase-3 immunohistochemistry. Neuropathology (H&E; frontal cortex).	10 min	Immunohistochemistry score (mean ± SEM): no ILE (217.8 ± 3.4); ILE (189.0 ± 3.1; $p < 0.05$). Neuropathology score (mean ± SD): no ILE (37.5 ± 3.7); ILE (33.8 ± 3.1; $p > 0.05$).	Scoring system used was poorly defined. Comparison of means performed post hoc using MedCalc[1]
Branco 2021 [54]	Rabbit	Ivermectin	Scored neurological examination, brain histopathology, brain ivermectin concentration.	6 h	Mean neurological score at 6 h: ILE (-): 9.75; (+ILE): 11.80 (NSD). Neuropathology: not observed. Mean (± SD) ivermectin concentration ILE (-): 0.36 ± 0.26 mg/kg; ivermectin (+ILE): 0.48 ± 0.18 mg/kg (NSD).	Increased ALT activity and serum triglyceride concentration in rabbits receiving ILE. Ivermectin concentration and histopathology of other tissues were performed. Additional treatment groups not included here. Limited neuropathology performed.

https://doi.org/10.3390/xxxxx

Branco 2023 [55]	Rabbit	Diazepam	Neurological examination	100 min	Mean neurological score at 10 min: ILE (-): 51; (+ILE): 48.5 (NSD) at 100 min: ILE (-): 2; (+ILE): 3.5 (NSD)	Additional treatment groups not included here.
Brand 2008 [56]	Mouse	Delta-9-tretrahydro-cannabinol	Rota-rod running time (3 cm, 16 rpm). and analgesia by tail-flick latency (40 mW/cm^2)	4 h	THC did not affect the ability of the mice to stay on the rota-rod. Intralipid did not influence the analgesic (tail-flick) effect of delta-9- tretrahydrocannabinol.	Quantitative data was not provided for ILE data. Rota rod performance not provided. Additional propofol treatment groups not included here.
Celikel 2014 [28]	Rat	Malathion	Clinical observations, brain histopathology	8 h	Incidence of dizziness, paralysis (including hind- and fore-limb): ILE (-): 100%; ILE (+): 100% (NSD). Mean time to time to onset of signs (hindlimb paralysis): ILE (-): 57.5 min ILE (+): 63 min (NSD). Brain histopathology changes were reported however incidence or severity data in controls versus ILE-treated animals was not provided.	Blood pseudo-cholinesterase activity and cholesterol, and triglyceride levels were evaluated. Limited neuropathology performed. Additional groups given atropine and pralidoxime (+/- ILE) not included here (no additional benefit of ILE reported).
Cocilova 2019 [57]	Turtle	Brevetoxin (PbTx-3)	Video recorded clinical observations. Brain brevetoxin concentration.	1, 6, or 24 h	Signs at 6 hr: ILE (-): significant muscle twitching, circling, head bobbing, paralysis, ataxia; $ILE_{50\ mg/kg}$ (+): normal ambulation, slight lethargy; $ILE_{100\ mg/kg}$ (+): normal ambulation, no lethargy. Signs at 24 hr: ILE (-): some paralysis and ataxia, lethargy; ILE (+): normal ambulation, no lethargy. Brain brevetoxin concentration at 1 or 24 h (ILE (-) versus ILE (+): NSD.	Additional cholestyramine treatment groups and tissue brevetoxin concentrations not included here. Qualitative assessment of clinical signs only, Small group sizes (n = 2 to 4/treatment/time). ILE

						pre-treatment group not included here.
Ghadiri 2017 [32]	Rat	Methamphetamine	Locomotor activity, stereotyped behavior incidence, brain histopathology	Behavior 24 h. Brain histopathology with terminal deoxynucleotidyl transferase-mediated dUTP nick end labeling (TUNEL): 3 d	Freezing response: ILE (-) versus ILE (+): 0 to 2 h: NSD. Freezing response > 2 h: ILE (+) < ILE (-), $p < 0.05$. Rearing responses: 0 to 4 h: NSD. Number of rears > 4 h: ILE (+) > ILE (-), $p < 0.01$ to $< p < 0.05$. Locomotor activity: 0 to 4 h: NSD. Locomotor activity > 4 h: ILE (+) > ILE (-), $p < 0.01$ to $< p < 0.05$. Stereotyped behavior at 8 h only: ILE (+) < ILE (-), $p < 0.05$. Mean (± SEM brain TUNEL positive cells: ILE (+) = 2.1 ± 0.3 %; ILE (-) = 7.1 ± 0.3 %; $p < 0.05$.	Additional outcomes (lung) not reported here. TUNEL data estimated using plot digitizer software (DigitizeIt). Limited neuropathology performed.
Hori 2022 [58]	Rat	Sevoflurane	Time to restore righting reflex after or during anesthesia.	5 min	Mean time (± SD) restore righting reflex: ILE (+) = 131 ± 53 s; ILE (-) = 237 ± 69 s; $p < 0.05$.	Same animals used for all phases. Additional cardiovascular effects not reported here.
	Rat	Isoflurane	Time to restore righting reflex after anesthesia.	5 min	Mean time (± SD) to restore righting reflex: ILE (+) = 203 ± 111 s; ILE (-) = 314 ± 154 s; $p < 0.05$.	
Jesmok 1979 [59]	Rat	Pentobarbital	Sleep time	200 min	Mean sleep time (± SE): $ILE_{2.5\ g/kg}$: 87 ± 6 min; ILE (-): 177 ± 15 min; $p < 0.001$.	Additional heparin treatment groups not included here. Figure 1 does not match text – and data has not been included.
Kayipmaz 2014 [60]	Rat	Methyl parathion	Clinical observations, brain electron microscopy	12 h	ILE decreased expansion of glial feet involved in the structure of the blood-brain barrier when compared to ILE(-) rats. Clinical observations: incidence of tremors: ILE(+): 4/7; ILE(-): 1/7; irritability: ILE(+): 4/7; ILE(-): 2/7 (NSD).	All rats treated with atropine and pralidoxime chloride. Data from other groups (no atropine and pralidoxime chloride) not included here. Limited

						neuropathology, brain samples poorly described, qualitative assessment only
Li 2012 [38]	Rat	Dichlorvos	Clinical observations	24 h	Clinical observations: incidence of salivation: ILE(+): 8/12; ILE(-): 11/12 ($p = 0.28$). Mean (± SD) muscle tremor intensity score: ILE(+) = 1.95 ± 0.25; ILE(-): 2.58 ± 0.19 (NSD).	Salivation incidence in manuscript was reported to be $p < 0.05$. Data from other groups (atropine and pralidoxime chloride) not included here.
Moshiri 2013 [42]	Rat	Diazinon	Clinical observations	48 h	Clinical observations: diarrhea score, muscle tone, fasciculations, and convulsions: NSD	Data from other groups not included here
Moshiri 2014 [43]	Rabbit	Haloperidol	Clinical observations and neuropathology	24 h	Clinical observations: Miosis at 1 h: ILE6 (+) (NSD), ILE12(+) and ILE18(+) decreased. Cataleptic score (malpositioned forelimb on back) at 2 h increased ($p < 0.05$) with ILE12(+) and ILE18(+). At 3 h cataleptic score increased ($p < 0.001$) with ILE6 (+), ILE12(+) and ILE18(+). Cataleptic score (malpositioned forelimb) decreased at 3 h with ILE6 (+), ILE12(+) and ILE18(+). Cataleptic score (standing time) decreased ($p < 0.001$) at 2 h with ILE6 (+), ILE12(+) and ILE18(+). Brain histopathology with TUNEL staining: NSD.	Mortality rate was significantly higher in rabbits given haloperidol and ILE at 18 mL/kg (ILE18). Limited neuropathology, brain samples poorly described, quantitative data not provided.
Ozkan 2014 [61]	Rat	Chlorpyriphos	Brain histopathology	NR	Neurodegeneration score: ILE (+) = 32/70; ILE (-) = 37/70 (NSD). Number of apoptotic neurons: ILE (+) = 108; ILE (-) = 147 (decreased; p value not provided).	Limited neuropathology, brain samples poorly described, limited quantitative data provided, time from

						exposure to outcome assessment was not provided. Apoptosis evaluated using caspase-3, bcl-2 and Bax staining. Data from other groups (caffeic acid phenethyl ester) not included here.
Vahabzadeh 2013 [48]	Rabbit	Tramadol	Seizure incidence and severity	24 h	Mean (± SEM) seizure duration: ILE12 (+) = 24.40 ± 17.80 s ($p < 0.01$); ILE18 (+) = 58.20 ± 15.99 s; ILE(-) = 61.20 ± 9.67 s. Seizure duration in ILE12(+) decreased versus ILE(-); ($p < 0.01$.) Seizure incidence ILE6 (+): 0/5 decreased versus ILE(-) ($p < 0.001$).	Data for ILE only (no tramadol) group was not included. Seizure incidence was not provided for most groups.
Yousefsani 2019 [51]	Rat	Clozapine	Rotarod performance, seizure score	24 h	Seizure score: ILE (+) < ILE(-) from 0.75 to 1.25 h ($p < 0.05$). Time on rotarod: ILE > ILE(-) from 3 to 24 h ($p < 0.01$).	Unclear whether ILE(+) had reduced seizure scores after 1.5 h. Histopathology findings of lung and pancreas not reported here.

Abbreviations: NR: not reported; NSD: no significant difference; PO: per os; rpm: revolutions per min; SQ: subcutaneous; TUNEL: terminal deoxynucleotidyl transferase-mediated dUTP nick end labeling

https://doi.org/10.3390/xxxxx

Table S5. GRADE assessment of neurologic outcomes in animal toxicology studies evaluating ILE

Domain	Assessment	Rationale
Outcome: Neuropathology		
Risk of bias	Serious	Most studies lacked detailed reporting of randomization, allocation concealment, blinding of histopathology assessment. Several studies did not provide complete quantitative outcome data or statistical comparisons. Histopathologic scoring methods were often poorly standardized or incompletely described.
Inconsistency	Serious	Results were heterogeneous across toxicants, species, outcome measures, and follow-up intervals. Some studies demonstrated reductions in apoptotic markers following ILE treatment, whereas others showed no significant differences in neurodegeneration or histopathology scores. Direction and magnitude of effect varied.
Indirectness	Serious	Neuropathologic surrogate markers such as caspase-3 immunohistochemistry or TUNEL staining may not directly translate to clinically meaningful neurologic outcomes. Exposure paradigms and timing of ILE administration may not reflect real-world poisoning scenarios.
Imprecision	Serious	Sample sizes were small in most studies, confidence intervals were generally not reported, and several studies lacked quantitative data entirely. Short follow-up periods limited assessment of neuropathologic outcomes. Many analyses were underpowered to detect clinically meaningful effects.
Publication bias	Possible	The evidence base consists primarily of small experimental studies with positive or exploratory findings. Negative or neutral animal studies may be underreported.
Outcome: Neurologic signs		
Risk of bias	Serious	Most studies lacked detailed reporting of randomization, allocation concealment, and blinding of neurologic assessment. Neurologic scoring systems were inconsistently described and often subjective. Several studies relied on observational clinical assessments.
Inconsistency	Serious	Findings varied substantially across toxicants and neurologic endpoints. Some studies demonstrated reduced seizure activity or improved neurologic signs with ILE, whereas many others showed no significant differences. Magnitude and direction of effect were inconsistent.
Indirectness	Possible	Many neurologic endpoints relied on observational findings that may not directly predict clinically meaningful neurologic recovery in animals.
Imprecision	Serious	Most studies included small sample sizes with limited statistical power. Confidence intervals were rarely reported, and several outcomes lacked complete quantitative data. Short monitoring periods further limited assessment of sustained neurologic recovery.

https://doi.org/10.3390/xxxxx

Publication bias	Possible	The evidence base consists primarily of small experimental studies, increasing the possibility of selective publication of positive findings while neutral or negative studies remain unpublished.
Outcome: Behavior		
Risk of bias	Serious	Studies provided limited information regarding randomization, allocation concealment, and blinding of behavioral assessment. Behavioral testing methods were variably described, and observer bias may have influenced outcome assessment in some studies.
Inconsistency	Serious	Behavioral outcomes differed substantially across toxicants and endpoints. One study demonstrated no significant effect, whereas others reported improvement in selected behavioral measures. Direction and magnitude of effect varied depending on time point and behavioral domain assessed.
Indirectness	Possible	Behavioral tests such as rotarod performance, tail-flick latency, and locomotor activity may not directly translate to clinically meaningful neurologic or functional recovery in animals.
Imprecision	Serious	The evidence base consisted of only two small studies with limited statistical power. Confidence intervals were not reported, and several findings were based on multiple exploratory behavioral comparisons across time points.
Publication bias	Possible	The small number of experimental studies and predominance of positive exploratory findings raise concern for selective publication and underreporting of neutral or negative studies.
Outcome: Recovery from anesthesia		
Risk of bias	Serious	Studies provided limited methodological detail regarding randomization, allocation concealment, and blinding of investigators. Although outcomes such as righting reflex and sleep duration are relatively objective, incomplete reporting limits confidence in internal validity.
Inconsistency	Not serious	All studies consistently demonstrated faster recovery from anesthesia or sedation following ILE administration across different anesthetic agents and outcome measures. Direction of effect was uniform.
Indirectness	Serious	Evidence focused on surrogate anesthetic recovery endpoints such as righting reflex restoration and sleep duration. These findings may not directly translate to clinically meaningful neurologic recovery or toxicologic outcomes in animals.
Imprecision	Serious	The evidence base consisted of only two small studies with limited sample sizes and incomplete reporting of confidence intervals or power calculations.
Publication bias	Suspected	The small number of experimental studies and predominance of positive findings raise concern for selective publication bias. Negative or neutral studies may be underreported.

Table S6. Excluded studies and reasons for exclusion

Citation	Reason for exclusion
Anonymous. IV fat emulsion for the treatment of ivermectin toxicosis. Adv. Small Anim. Med. Surg. 2012;25(7):4-5.	No original data
Committee on Toxicology Laboratories. [Serial publication from the Committee on Toxicology Laboratories--Application of parenteral lipid emulsions to therapy for poisonings]. Chudoku Kenkyu. 2009 Mar;22(1):70-4. Japanese.	No original data
Anonymous. Parenteral nutrition in the newborn--a time for caution. Lancet 1980;2(8199):838-839.	No original data
Heird WC, et al. Structured lipids and their use in clinical nutrition. Am J Clin Nutr 1986;43(2):320-324	No original data
Nugent KM. Intralipid effects on reticuloendothelial function. J Leukoc Biol. 1984 Aug;36(2):123-32.	No original data
Adachi YU, Numaguchi A, Matsuda N. Not only bupivacaine but also propofol is sinking in lipid? J Anesth. 2014 Jun;28(3):476. doi: 10.1007/s00540-013-1725-2.	No original data
Akyol BA, Gokbulut C. The effect of intravenous lipid emulsion (ILE) on the pharmacokinetic/toxicokinetic dispositions of ivermectin and carprofen in rabbits. Naunyn Schmiedebergs Arch Pharmacol. 2024 Mar;397(3):1841-1852.	Wrong outcomes
Alp, H; Pinar, N; Dokuyucu, R; et al. Effects of intralipid and caffeic acid phenyl esther (CAPE) against hepatotoxicity and nephrotoxicity caused by glyphosate isopropylamine (GI). Eur J Inflamm. 2016;14(1):3-9	Wrong outcomes
Alp H, Pinar N, Dokuyucu R, et al. Protective effects of intralipid and caffeic acid phenethyl ester (CAPE) on hepatotoxicity and pancreatic injury caused by dichlorvos in rats. Biochem Genet. 2016 Dec;54(6):803-815.	Wrong outcomes
Arcuri E, Bellelli L, Aloe L. L'alimentazione lipidica in oncologia [Lipid feeding in oncology]. Minerva Anestesiol. 1981 Jul;47(7):359-61. Italian.	Wrong outcomes
Atik M. Platelet dysfuction after intravenous infusion of fat emulsion. Acta Chir Scand Suppl. 1976;466:68-9.	Wrong study design
Atik M, Marrero R, Isla F, Manale B. Hemodynamic changes following infusion of intravenous fat emulsions. Am J Clin Nutr. 1965 Jan;16:68-74.	Wrong outcomes
Awad MT, Alkhathlan M, Spetz SL, Cet al. Intravenous Lipid Emulsion as Salvage Treatment for Haloperidol Toxicity. Am J Ther. 2020 Jan 22;28(5):e612-e614.	Reply to editor
Ayan M, Tas U, Sogut E, et al. Effect of Intravenous Lipid Emulsion Therapy on Serum Pseudocholinesterase in Experimental Model of Diazinon Intoxication. Eur J Gen Med 2014;11(4):268-71.	Wrong outcomes

Banerjee S, Zargari M, Medzikovic L, et al. Intralipid® improves left ventricular function in rats with lipopolysaccharide-induced endotoxaemia by a Src-STAT3-mediated mechanism. Br J Anaesth. 2023 Feb;130(2):e183-e187.	Wrong outcomes
Bates N, Edwards N. Use of intravenous lipid emulsion in poison cases. Veterinary Times 01/01/2015 2015;45(23):19	No original data
Bates N. Lipid infusion in the management of poisoning. Vet Rec 2016;179(1):22	No original data
Bates N, Edwards N. Glyphosate toxicity in animals. Clin Toxicol (Phila). 2013 Dec;51(10):1243.	No original data
Bazin, Y; Bresson, D; Montharu, J; et al. Interest in lipid emulsion infusion during acute digoxin intoxication in rats. Ann Fr Anesth Reanim. 2013;32():A383-A383	Proceeding
Berry IM, Ivy AC. The tolerance of dogs to intravenously administered fatty chyle and synthetic fat emulsion. Fed Proc. 1948 Mar;7(1 Pt 1):7.	Wrong outcomes
Biber B, Martner J, Werner O. Halothane by the I.V. route in experimental animals. Acta Anaesthesiol Scand. 1982 Dec;26(6):658-9.	Wrong study design
Biozzi G, Stiffel C, Mouton D. [Stimulation and depression of the phagocytic function of the reticuloendothelial system by lipid emulsions. Relation to some immunological phenomena]. Rev Fr Etud Clin Biol. 1963 Apr;8:341-57. French.	Wrong outcomes
Black CD, Popovich NG. Stability of intravenous fat emulsions. Arch Surg. 1980 Jul;115(7):891.	No original data
Bora S, Erdoğan MA, Yiğittürk G, et al. The effects of lipid emulsion, magnesium sulphate and metoprolol in amitriptyline-induced cardiovascular toxicity in rats. Cardiovasc Toxicol. 2018 Dec;18(6):547-556.	Wrong outcomes
Brent J. A systematic review of the use of intravenous lipid therapy in the management of poisoned patients. Clin Toxicol 2010;48(3):241-242	No original data
Brent J. Poisoned patients are different--sometimes fat is a good thing. Crit Care Med. 2009 Mar;37(3):1157-8.	No original data
Browne A, Harvey M, Cave G. Intravenous lipid emulsion does not augment blood pressure recovery in a rabbit model of metoprolol toxicity. J Med Toxicol. 2010 Dec;6(4):373-8.	Wrong outcomes
Bruenisholz H, Kupper J, Muentener CR, et al. Treatment of ivermectin overdose in a miniature Shetland Pony using intravenous administration of a lipid emulsion. J Vet Intern Med. 2012 Mar-Apr;26(2):407-11.	Case report
Bryant MS, Sinclair KG, Copeland EM 3rd, et al. Absorption of a parenteral diet from the peritoneal cavity. Curr Surg. 1987 Sep-Oct;44(5):382-4.	Wrong study design
Butterfield S. Clinical approach to the acute neurological intoxication. Vet Ireland J 01/01/2022 2022;12(5):262-267,	No original data

Calder PC. More good news about fish oil. Nutrition. 2001 Feb;17(2):158-60.	No original data
Calder PC, Deckelbaum RJ. 'New' metabolic pathways and roles for lipids and lipid emulsions. Curr Opin Clin Nutr Metab Care. 1998 Mar;1(2):139-41.	No original data
Carey A, Duggan CP. Essentially well tolerated: a novel way to examine fatty acid status in children with severe intestinal failure on composite fish-oil lipid emulsion. Am J Clin Nutr. 2022 Feb 9;115(2):332-333.	Wrong study design
Carlson LA, Hallberg D. Studies on the elimination of exogenous lipids from the blood stream. The kinetics of the elimination of a fat emulsion and of chylomicrones in the dog after single injection. Acta Physiol Scand. 1963;59():52-61.	Wrong study design
Carreiro S, Hack JB, Blum J. In reply. Ann Emerg Med. 2015 Jan;65(1):127-8.	No original data
Carreiro S, Hack JB, Blum J. In reply. Ann Emerg Med. 2015 Jan;65(1):127-8.	Reply to editor
Cave G, Harvey M. Intralipid infusion in rabbit asphyxial pulseless electrical activity: a pilot study. Eur J Anaesthesiol. 2008 May;25(5):428-30.	Wrong outcomes
Cave G, Harvey M, Quinn P, Heys D. Hypertonic sodium bicarbonate versus intravenous lipid emulsion in a rabbit model of intravenous flecainide toxicity: no difference, no sink. Clin Toxicol (Phila). 2013 Jun;51(5):394-7.	Wrong outcomes
Cave G, Harvey M. Lipid emulsion may augment early blood pressure recovery in a rabbit model of atenolol toxicity. J Med Toxicol. 2009 Mar;5(1):50-1. doi: 10.1007/BF03160983.	Wrong outcomes
Cave G, Harvey MG, Castle CD. Intralipid ameliorates thiopentone induced respiratory depression in rats: investigative pilot study. Emerg Med Australas. 2005 Apr;17(2):180-1.	Wrong outcomes
Celikmen, MF; Sarikaya, S; Özücelik, DN; et al. Comparison of Normal saline, Activated Charcoal and Intravenous Lipid Emulsion in a Rat Model of Colchicine Overdose: Experimental Study. Indian J. Pharm. Educ. Res. 2024;58(3):794-801	Wrong outcomes
Chalifoux NV, Butty EM, Mauro KD, et al. Outcomes of 434 dogs with non-steroidal anti-inflammatory drug toxicosis treated with fluid therapy, lipid emulsion, or therapeutic plasma exchange. J Vet Intern Med. 2023 Jan;37(1):161-172.	Wrong study design
Charlton NP, Morse PS, Borek HA, et al. The toxicology literature of 2011: issues impacting the emergency physician. Am J Emerg Med. 2013 Mar;31(3):597-601.	No original data
Chen BC, Taylor M, Langevin M, Hoffman RS. Should transient physiologic effects following lipid administration be attributed to a cellular signaling mechanism without cellular data? Crit Care Med. 2013 Nov;41(11):e389.	No original data
Chen WJ. Effects of sepsis on the rates of plasma clearance and CO2 production of intravenously administered intralipid emulsion in rats. Taiwan Yi Xue Hui Za Zhi. 1983 Feb;82(2):203-12.	Wrong study design
Chen WJ. Utilization of exogenous fat emulsion at early stage of injury in rats. Taiwan Yi Xue Hui Za Zhi. 1984 May;83(5):413-8.	Wrong outcomes

Chen WJ, Lin JY, Tai TY, Chen CC, Hung WT. Total parenteral nutrition employing fat emulsion--a clinical and experimental study. Taiwan Yi Xue Hui Za Zhi. 1979 Feb;78(2):70-86.	Wrong intervention
Cuthbertson D. Historical background to parenteral nutrition. Acta Chir Scand Suppl. 1980;498:1-11.	No original data
Dalgleish D, Katawaroo S. Lipid emulsion to treat bupivacaine toxicity. Anaesthesia. 2005 Aug;60(8):822.	No original data
Davis SS, Washington C, West P, et al. Lipid emulsions as drug delivery systems. Ann N Y Acad Sci 1987;507():75-88	No original data
Davis W. Factors related to recovery in a cat treated for lidocaine intoxication. J Am Vet Med Assoc. 2011 Feb 1;238(3):284	Reply to editor
Di Gregorio G, Schwartz D, Ripper Ret al. Lipid emulsion is superior to vasopressin in a rodent model of resuscitation from toxin-induced cardiac arrest. Crit Care Med. 2009 Mar;37(3):993-9.	Wrong outcomes
Doğanay F, Ak R, Alışkan H, et al. The Effects of Intravenous Lipid Emulsion Therapy in the Prevention of Depressive Effects of Propofol on Cardiovascular and Respiratory Systems: An Experimental Animal Study. Medicina (Kaunas). 2018 Dec 25;55(1):1.	Wrong study design
Dokuyucu R, Bilgili A, Hanedan B, et al. Attenuating effects of caffeic acid phenethyl ester with intralipid on hepatotoxicity of chlorpyrifos in the case of rats. Med Pr. 2016 Dec 22;67(6):743-749.	Wrong outcomes
Driscoll DF. Regarding "A Comparison of Fish Oil Sources for Parenteral Lipid Emulsions in a Murine Model". JPEN J Parenter Enteral Nutr. 2017 Jul;41(5):703.	Reply to editor
du Toit DF, Villet WT. Fat infusions in clinical practice. S Afr Med J. 1978 Oct 28;54(18):730.	No original data
El-Bahri L. Role of IV lipid emulsion antidote. Veterinary Times 01/01/2016 2016;46(12):9-10	No original data
Elnaggar AM, Khorshid OA; et al. Muscle response of anticholinesterase-intoxicated rats to different therapeutic modalities. Egypt J Neurol Psychiatr Neurosurg 2015;52(3):206-211.	Wrong study design
Enokiya T, Zhang E, Ikemura K, et al. Effect of lipid emulsion infusion on paliperidone pharmacokinetics in the acute overdose rat model: A potential emergency treatment for paliperidone intoxication. Eur J Pharm Sci. 2017 Nov 15;109:217-222.	Wrong outcomes
Esen M, Uysal M. Protective effects of intravenous lipid emulsion on malathion-induced hepatotoxicity. Bratisl Lek Listy. 2018;119(6):373-378.	Wrong outcomes
Fettiplace MR. Reply to Hoegberg et al.: letter in response to "lipid emulsion improves survival in animal models of local anesthetic toxicity: a meta-analysis". Clin Toxicol (Phila). 2017 Nov;55(9):1021-1022.	Reply to editor
Fettiplace MR, Weinberg G. The authors reply. Crit Care Med. 2013 Nov;41(11):e389-90.	Reply to editor

Fettiplace MR, Weinberg G, Ripper R, et al. Intraosseous Injection of Intralipid Reverses Bupivacaine-Induced Cardiovascular Toxicity in Rats. Am J Respir Crit Care Med. 2013; 187 A4410.	Wrong outcomes
Fischer GW, Hunter KW, Wilson SR. Intralipid and reticuloendothelial clearance. Lancet. 1980 Dec 13;2(8207):1300.	Reply to editor
Fürst P, Kuhn KS. Fish oil emulsions: what benefits can they bring? Clin Nutr. 2000 Feb;19(1):7-14.	No original data
Goor Y, Goor O. Has the silver bullet been found? Reg Anesth Pain Med. 2004 Jan-Feb;29(1):73-4	No original data
Gordon S. Does soybean intravenous lipid emulsion therapy reduce time to recovery faster than ClinOleic therapy in cats with permethrin toxicosis? Vet Evid 2021;6(4)	Review
Gould DB. Halogenated gas embolism prevention by Intralipid. Can J Anaesth. 1995 Sep;42(9):836-7.	Reply to editor
Greenberg D. Fats and satiety: the role of the small intestine. Appetite. 1998 Oct;31(2):229.	No original data
Grossman MI, Strub IH. Effect of heparin on fate of intravenously administered fat emulsions in rats. Proc Soc Exp Biol Med. 1954 Feb;85(2):356-9.	Wrong study design
Gueret G, Pennec JP, Arvieux CC. Hemodynamic effects of intralipid after verapamil intoxication may be due to a direct effect of fatty acids on myocardial calcium channels. Acad Emerg Med. 2007 Aug;14(8):761.	Reply to editor
Gwaltney-Brant SM, DeClementi C, Gupta RC. Macrocyclic Lactone Endectocides. Veterinary Toxicology (Third Edition), 2018, Pages 539-550.	No original data
Hallberg D. Therapy with fat emulsion. Acta Anaesthesiol Scand Suppl. 1974;55:131-6.	No original data
Harvey M, Cave G, Hoggett K. Correlation of plasma and peritoneal diasylate clomipramine concentration with hemodynamic recovery after intralipid infusion in rabbits. Acad Emerg Med. 2009 Feb;16(2):151-6.	Wrong outcomes
Harvey M, Cave G, Lahner D, et al. Insulin versus Lipid Emulsion in a Rabbit Model of Severe Propranolol Toxicity: A Pilot Study. Crit Care Res Pract. 2011;2011:361737.	Wrong outcomes
Harvey M, Cave G, Shaw T. Effect of intravenous lipid emulsion and octreotide on enteric thiopentone absorption; a pilot study. Clin Toxicol (Phila). 2013 Feb;51(2):117-8.	Wrong study design
Harvey MG, Cave GR. Intralipid infusion ameliorates propranolol-induced hypotension in rabbits. J Med Toxicol. 2008 Jun;4(2):71-6.	Wrong outcomes
Harvey M, Cave G. Lipid rescue: does the sink hold water? And other controversies. Br J Anaesth. 2014 Apr;112(4):622-5.	No original data

Hatae T. Electron microscopic observations on the mouse liver after intravenous administration of fat emulsion. Arch Histol Jpn. 1974 Sep;37(2):149-63.	Wrong outcomes
Henwood S, Wilson D, White R, Trimbo S. Developmental toxicity study in rats and rabbits administered an emulsion containing medium chain triglycerides as an alternative caloric source. Fundam Appl Toxicol. 1997 Dec;40(2):185-90.	Wrong outcomes
Hoegberg LCG, Hoffman RS, Hayes BD, Lavergne V, Gosselin S. Letter in response to "Lipid emulsion improves survival in animal models of local anesthetic toxicity: a meta-analysis". Clin Toxicol (Phila). 2017 Nov;55(9):1019.	Reply to editor
Höjer J, Jacobsen D, Neuvonen PJ, Rosenberg PH. Lipid Rescue - Efficacy and Safety Still Unproven. Basic Clin Pharmacol Toxicol. 2016 Oct;119(4):345-8.	No original data
Hori K, Kuno M, Nishikawa K. Lipid emulsion increases the fast Na+ current and reverses the bupivacaine-induced block: a new aspect of lipid resuscitation? Anesthesiology. 2014 Oct;121(4):903-4.	Reply to editor
Hunt CE, Gora P, Inwood RJ. Pulmonary effects of Intralipid: the role of Intralipid as a prostaglandin precursor. Prog Lipid Res. 1981;20:199-204.	Wrong study design
Jeppsson R. Comparison of pharmacological effects of some local anaesthetic agents when using water and lipid emulsion as injection vehicles. Acta Pharmacol Toxicol (Copenh). 1975 Apr;36(4):299-311.	Wrong outcomes
Jourdan G, Boyer G, Raymond-Letron I, et al. Intravenous lipid emulsion therapy in 20 cats accidentally overdosed with ivermectin. J Vet Emerg Crit Care (San Antonio). 2015 Sep-Oct;25(5):667-71.	Wrong study design
Jurewitsch B. The evolving utility and emergent uses of novel lipid emulsions: new horizons and rediscovered prospects. JPEN J Parenter Enteral Nutr. 2012 Nov;36(6):626-9.	No original data
Kazemi A, Harvey M, Cave G, Lahner D. The effect of lipid emulsion on depth of anaesthesia following thiopental administration to rabbits. Anaesthesia. 2011 May;66(5):373-8.	Wrong intervention
Kennedy MJ, Smith LJ. Anesthesia case of the month. Accidental lidocaine overdose during surgery for vertebral fractures. J Am Vet Med Assoc. 2014 Nov 15;245(10):1098-101.	Case report
Killoran PV, Cattano D. From bedside to bench and back: perfecting lipid emulsion therapy for local anesthetic toxicity. Anesthesiology. 2011 Dec;115(6):1151-2.	No original data
Kim DK, Pfeifer J. Measurement of phagocytic activity of reticuloendothelial system (RES) by Intralipid: effect of C parvum treatment. Surg Forum. 1977;28:85-7.	Wrong outcomes
Li G, Hu H. Protective effects of lipid emulsion on vital organs through the LPS/TLR4 pathway in acute organophosphate poisoning. BMC Pharmacol Toxicol. 2025 Mar 27;26(1):71. doi: 10.1186/s40360-025-00904-4.	Wrong outcomes
Litonius E, Niiya T, Petäjä L, et al. Lipid resuscitation from amiodarone overdose in pigs. Eur J Anaesth 2010;27(1):190-191.	Wrong outcomes
Liu X, Zhang J, Zhao H, et al. The effect of propofol on intrathecal morphine-induced pruritus and its mechanism. Anesth Analg. 2014 Feb;118(2):303-309.	Wrong outcomes

Margarido NF, Lima-Gonçalves E, Bevilacqua RG, et al. [Exclusive parenteral feeding in partially restrained rats. II. Comparative evaluation of the use of aminoacid solutions and lipid emulsions]. Rev Paul Med. 1979 Jul-Aug;94(1-2):11-8.	Wrong study design
Martin TG. Intravenous lipid emulsion therapy for poisonings. Clin Toxicol. 2008;46(5):403-403	No original data
Mathieu S, Cranshaw J. Treatment of severe local anaesthetic toxicity. Anaesthesia. 2008 Feb;63(2):202-3; author reply 203.	No original data
Mayr VD, Raedler C, Wenzel V, et al. Lipid, not propofol, treats bupivacaine overdose. Anesth Analg. 2004 Dec;99(6):1876.	Reply to editor
Moshiri M, Vahabzadeh M, Mohammadpour AH, Hosseinzadeh H. Evaluation of intravenous lipid emulsion on haloperidol-induced hypotension in rabbits. Toxicol Ind Health. 2016 May;32(5):945-52.	Wrong outcomes
Oda Y. Lipid resuscitation: development in basic research and application to clinical practice. J Anesth. 2013 Dec;27(6):811-4.	No original data
Ozcan MS, Weinberg G. Update on the use of lipid emulsions in local anesthetic systemic toxicity: a focus on differential efficacy and lipid emulsion as part of advanced cardiac life support. Int Anesthesiol Clin. 2011 Fall;49(4):91-103.	No original data
Ozen SB, Celebi SH, Coskun D, Gungor I, Inan G. Effect of intralipid administration on calcium therapy in verapamil toxicity. Bratisl Lek Listy. 2019;120(10):789-793.	Wrong study design
Perez E, Bania TC, Medlej K, Chu J. Determining the optimal dose of intravenous fat emulsion for the treatment of severe verapamil toxicity in a rodent model. Acad Emerg Med. 2008 Dec;15(12):1284-9.	Wrong outcomes
Perichon D, Turfus S. Graudins A. Intravenous lipid emulsion does not improve hemodynamics or survival in a rodent model of oral verapamil poisoning. Clin Toxicol 2013;51(4):277-277	Wrong outcomes
Perrault JR, Barron HW, Malinowski CR, Milton SL, Manire CA. Use of intravenous lipid emulsion therapy as a novel treatment for brevetoxicosis in sea turtles. Sci Rep. 2021 Dec 17;11(1):24162.	Wrong study design
Picard J, Harrop-Griffiths W. Lipid emulsion to treat drug overdose: past, present and future. Anaesthesia. 2009 Feb;64(2):119-21.	No original data
Picard J, Meek T. Lipid emulsion to treat overdose of local anaesthetic: the gift of the glob. Anaesthesia. 2006 Feb;61(2):107-9.	No original data
Putić V, Jović-Stošić J. Intravenous fat emulsion in clinical practice: nutrient and antidote. Vojnosanit Pregl. 2015 Mar;72(3):274-9.	No original data
Rosenberg PH. Current evidence is not in support of lipid rescue therapy in local anaesthetic systemic toxicity. Acta Anaesthesiol Scand. 2016 Sep;60(8):1029-32.	No original data
Rowlingson JC. Lipid rescue: a step forward in patient safety? Likely so! Anesth Analg. 2008 May;106(5):1333-6.	No original data

Schediwy M, Mevissen M, Demuth D, et al. [New causes of animal poisoning in Switzerland]. Schweiz Arch Tierheilkd. 2015 Mar;157(3):147-52. German.	No original data
Shils ME. Recalling a 63-year nutrition odyssey. Nutrition. 2000 Jul-Aug;16(7-8):582-5.	No original data
Stewart R, Newman V, Harvey M, et al. Lipid emulsion mitigates intravenous amiodarone toxicity in a rat model. Toxicol Comm 2022;6(1), 30–34.	Wrong out-comes
Tanioka M, Park WK, Shim I, et al. Neuroprotection from Excitotoxic Injury by Local Administration of Lipid Emulsion into the Brain of Rats. Int J Mol Sci. 2020 Apr 14;21(8):2706.	Wrong study design
Tasci A, Tasci EDK, Turan CA, Ozturk TC. Acute Pioglitazone Intoxication in Rats: Lack of Protective Effect of Intravenous Lipid Emulsion-A Pilot Study. J Appl Toxicol. 2026 Mar;46(3):923-931; Epub 2025 Sep 22.	Wrong out-comes
Tasci EDK, Ozturk TC, Kocak M, et al. Investigation of Toluene Toxicity in an Animal Model: The Role of Intralipid Emulsion. J Appl Toxicol. 2026 Jan;46(1):229-238; Epub 2025 Aug 10.	Wrong out-comes
Tebbutt S, Harvey M, Nicholson T, Cave G. Intralipid prolongs survival in a rat model of verapamil toxicity. Acad Emerg Med. 2006 Feb;13(2):134-9. doi: 10.1197/j.aem.2005.08.016.	Wrong out-comes
Tegner C, Lundgren S, Dreimanis K, et al. Alpha-chloralose poisoning in cats: treatment with an intravenous lipid emulsion. BSAVA Congress Proceedings 2023.	Proceeding
Tikhomirov M, Antonczyk A, Sniegocki T, et al. Intravenous lipid emulsion as a treatment in buprenorphine overdose - pharmaco-kinetic and pharmacodynamic evaluation in the rabbit model. Toxicol Lett 2021;350:S81-S81	Wrong out-comes
Tulgar S, Kose HC, Demir Piroglu I, et al. Comparison of Effects of Separate and Combined Sugammadex and Lipid Emulsion Administration on Hemodynamic Parameters and Survival in a Rat Model of Verapamil Toxicity. Med Sci Monit. 2016 Mar 25;22:984-90.	Wrong out-comes
Umar S, Nadadur RD, Li J, et al. Intralipid prevents and rescues fatal pulmonary arterial hypertension and right ventricular failure in rats. Hypertension. 2011 Sep;58(3):512-8.	Wrong out-comes
Uysal M, Karaman S. In vivo effects of intravenous lipid emulsion on lung tissue in an experimental model of acute malathion intoxication. Toxicol Ind Health. 2018 Feb;34(2):110-118.	Wrong out-comes
Uzunget SC, Evrin T, Uzunget SB, et al. Evaluation of activated charcoal and lipid emulsion treatment in model of acute rivaroxaban toxicity. Am J Emerg Med. 2018 Aug;36(8):1346-1349.	Wrong out-comes
Weinberg GL. Lipid emulsion infusion: resuscitation for local anesthetic and other drug overdose. Anesthesiology. 2012 Jul;117(1):180-7.	No original data
Weinberg GL, Bedocs P, Fettiplace MR. Pigs and Paradigms: Stop Using Swine to Study Lipid Resuscitation. Anesth Analg. 2019 Jul;129(1):4-7.	No original data

Weinberg G, Suresh S. Local anesthetic systemic toxicity and animal models for rescue paradigms: can pigs fly? Paediatr Anaesth. 2012 Feb;22(2):121-3.	No original data
Weinberg G, Warren L. Lipid resuscitation: listening to our patients and learning from our models. Anesth Analg. 2012 Apr;114(4):710-2.	No original data
Wright HM, Chen AV, Talcott PA, et al. Intravenous fat emulsion as treatment for ivermectin toxicosis in three dogs homozygous for the ABCB1-1Δ gene mutation. J Vet Emerg Crit Care (San Antonio). 2011 Dec;21(6):666-72.	Case report
Xanthos T, Psichalakis N, Russell D, et al. Intralipid™ administration attenuates the hypotensive effects of acute intravenous amiodarone overdose in a swine model. Am J Emerg Med. 2016 Aug;34(8):1389-93.	Wrong outcomes
Yamamoto K. [Current topics in local anesthesia]. Masui 2011;60 Suppl():S102-112	No original data
